# Targeting lonidamine to mitochondria mitigates lung tumorigenesis and brain metastasis

**DOI:** 10.1038/s41467-019-10042-1

**Published:** 2019-05-17

**Authors:** Gang Cheng, Qi Zhang, Jing Pan, Yongik Lee, Olivier Ouari, Micael Hardy, Monika Zielonka, Charles R. Myers, Jacek Zielonka, Katherine Weh, Andrew C. Chang, Guoan Chen, Laura Kresty, Balaraman Kalyanaraman, Ming You

**Affiliations:** 1 Free Radical Research Center, Medical College of Wisconsin, 8701 Watertown Plank Road, Milwaukee, WI 53226 USA; 2 Department of Biophysics, Medical College of Wisconsin, 8701 Watertown Plank Road, Milwaukee, WI 53226 USA; 3 Center for Disease Prevention Research, Medical College of Wisconsin, 8701 Watertown Plank Road, Milwaukee, WI 53226 USA; 4 Department of Pharmacology and Toxicology, Medical College of Wisconsin, 8701 Watertown Plank Road, Milwaukee, WI 53226 USA; 5 Aix Marseille Univ, CNRS, ICR UMR 7273, 13013 Marseille, France; 6 Section of Thoracic Surgery, Department of Surgery, Rogel Cancer Center, University of Michigan, 1500 East Medical Center Drive, Ann Arbor, MI 48109 USA

**Keywords:** Cancer, Oncology

## Abstract

Lung cancer often has a poor prognosis, with brain metastases a major reason for mortality. We modified lonidamine (LND), an antiglycolytic drug with limited efficacy, to mitochondria-targeted mito-lonidamine (Mito-LND) which is 100-fold more potent. Mito-LND, a tumor-selective inhibitor of oxidative phosphorylation, inhibits mitochondrial bioenergetics in lung cancer cells and mitigates lung cancer cell viability, growth, progression, and metastasis of lung cancer xenografts in mice. Mito-LND blocks lung tumor development and brain metastasis by inhibiting mitochondrial bioenergetics, stimulating the formation of reactive oxygen species, oxidizing mitochondrial peroxiredoxin, inactivating AKT/mTOR/p70S6K signaling, and inducing autophagic cell death in lung cancer cells. Mito-LND causes no toxicity in mice even when administered for eight weeks at 50 times the effective cancer inhibitory dose. Collectively, these findings show that mitochondrial targeting of LND is a promising therapeutic approach for investigating the role of autophagy in mitigating lung cancer development and brain metastasis.

## Abstract (web summary)

Brain metastases are a major reason for lung cancer mortality. Here, the authors modify lonidamine to target mitochondria, and show its therapeutic efficacy in inhibiting lung cancer brain metastasis by inducing autophagic cell death in cancer cells.

## Introduction

**Fig. 1:**
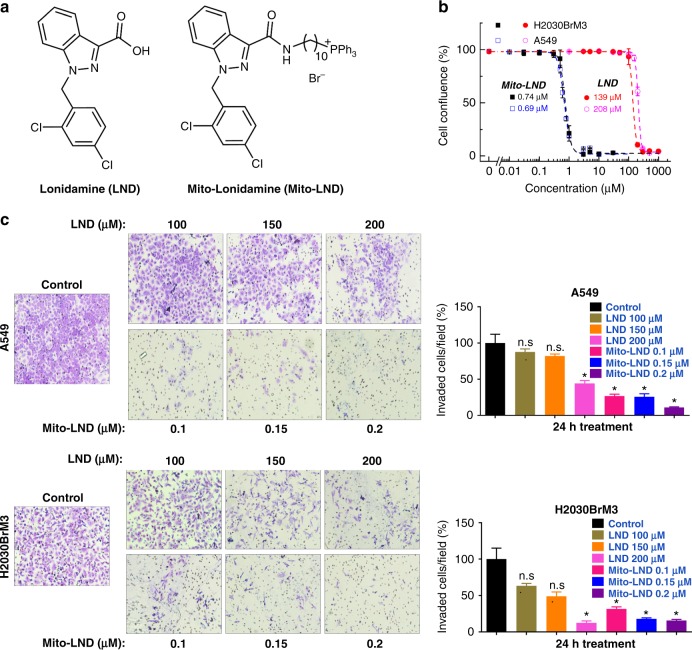
LND and Mito-LND structures and effects on proliferation and invasion. **a** Chemical structures of LND and Mito-LND. **b** Effect of LND and Mito-LND on the proliferation of human lung cancer cells. H2030BrM3 and A549 cells were treated with LND or Mito-LND. Cell proliferation was monitored in real-time with the continuous presence of indicated treatments until the end of each experiment. Dose response of LND and Mito-LND on cell confluence kinetics are shown in Fig. S4. The cell confluence (as control groups reach 98% confluency) is plotted against concentration. Dashed lines represent the fitting curves used to determine the IC_50_ values as indicated. **c**, **d** Effect of LND and Mito-LND on the invasion of brain metastatic human lung cancer cells. Representative figures from the transwell assay (H2030BrM3 and A549 cells) are shown in the left panels and quantitative analysis of the transwell data in the right panels. Data are presented as the means ± SEM. *t* test versus control: *p < 0.05; **p < 0.01

Non-small cell lung cancer (NSCLC) accounts for about 85% of lung cancer cases^1^. Brain metastases remain one of the leading causes of lung cancer mortality^2,3^. Current therapeutic options for brain metastases include whole brain/central nervous system irradiation, surgical resection, or treatment with small molecule tyrosine kinase inhibitors^4^. These therapies are essentially palliative and have significant risks and toxicity. New and effective chemotherapeutic drugs that prevent lung cancer progression and brain metastases are urgently needed. Lonidamine (LND) has undergone clinical trials in combination with standard-of-care chemotherapeutics for multiple cancers^5,6^. LND was previously evaluated in Phase II and Phase III trials targeting lung cancer and was shown to be safe, but to have limited efficacy^7^. LND (Fig. 1a) is a derivative of indazole-3-carboxylic acid and is known to inhibit aerobic glycolysis and energy metabolism selectively in tumor cells^8–10^. LND can also inhibit the succinate-ubiquinone reductase activity of respiratory complex II, leading to enhanced formation of reactive oxygen species (ROS)^11,12^.

Although tumor cells rely mostly on aerobic glycolysis to generate adenosine triphosphate (ATP) (the Warburg effect), many studies have shown that mitochondria are functional in most tumor cells^13–15^. Targeting mitochondrial bioenergetics is emerging as a viable approach to inhibit the growth of cancer cells^14,16,17^. Reports suggest that mitochondria-targeted cationic agents induce antiproliferative and cytotoxic effects in tumor cells but not in normal cells^18–20^. Mitochondria-targeted agents (e.g., Mito-Q and Mito-metformin) contain a triphenylphosphonium cation (TPP^+^) attached to various bioactive molecules (Co-Q and metformin)^20–22^. Linking the parent molecules to TPP^+^ via a long alkyl chain increases their lipophilicity and enhances cellular uptake^22–25^. Increased negative plasma membrane and mitochondrial transmembrane potentials in tumor cells facilitate the selective accumulation and retention of delocalized lipophilic cations^23,24^. LND has some potential mitochondrial mechanisms, so developing mitochondria-targeted lonidamine (Mito-LND) could markedly enhance its potency and/or efficacy and potentially identify additional mechanisms linking mitochondrial changes to cancer inhibition. To this end, we synthesized Mito-LND (Fig. 1a) by conjugating LND to TPP^+^ via a linker aliphatic chain, and its effects on lung cancer cells and lung cancer brain metastases are described.

Mito-LND is different in several aspects from other mitochondria-targeted cationic compounds^22^: Mito-LND appears to be one of the least toxic mitochondria-targeted cationic agents developed to date. In addition to inhibiting primary lung tumors, Mito-LND also suppresses lung cancer brain metastases in an orthotopic mouse model and is more effective than LND in both cases. Mito-LND induces autophagic cell death by inhibiting mitochondrial complexes I and II and stimulating ROS formation. These Mito-LND-mediated inhibitory effects on lung tumor cells occur at low micromolar concentrations that are readily achievable in vivo.

## Results

### Mito-LND blocks lung cancer growth, migration, and invasion

To compare the antiproliferative effects of LND and Mito-LND, human lung cancer cells were treated with LND or Mito-LND. Cell proliferation data show the concentration-dependent decrease in cell confluence kinetics of H2030BrM3 cells treated with LND and Mito-LND (Supplementary Fig. 1). The IC_50_ values for inhibiting cell proliferation were 188- and 300-fold lower for Mito-LND than LND for H2030BrM3 and A549 cells, respectively (Fig. 1b and Supplementary Fig. 1). In addition, attachment of the alkyl chain without the TPP moiety (Alkyl-LND) did not improve the potency of LND (Supplementary Fig. 2). Thus, linking LND to the TPP^+^ cation via an alkyl linker chain (Mito-LND) is essential to increase its antiproliferative potency. We confirmed the ability of Mito-LND to bind to isolated mitochondria in a membrane-potential-dependent manner; as shown in Supplementary Fig. 3, more Mito-LND was found outside mitochondria that had been treated with the mitochondrial uncoupler FCCP. Furthermore, to exclude the possibility that Mito-LND is metabolized to LND in cells, we tested the level of LND in cells treated with Mito-LND; since the level of LND was below the detection limit, LND levels were ≤0.1% of those of Mito-LND (Supplementary Fig. 4). To investigate the potential effects of Mito-LND and LND on lung cancer metastasis, we first tested their effects in vitro via the Boyden chamber invasion assay using H2030BrM3 brain metastasis and A549 lung cancer cell lines. Mito-LND (48 h treatment) was nearly 100-fold more potent than LND in suppressing cell invasion (Fig. 1c). Importantly, the anti-invasive effects of Mito-LND (Fig. 1c) were observed at lower concentrations than the antiproliferative effects (Fig. 1b); the anti-invasive effects cannot therefore be attributed to halting cell proliferation.

### Mito-LND inhibits mitochondrial complex I and II activities

**Fig. 2:**
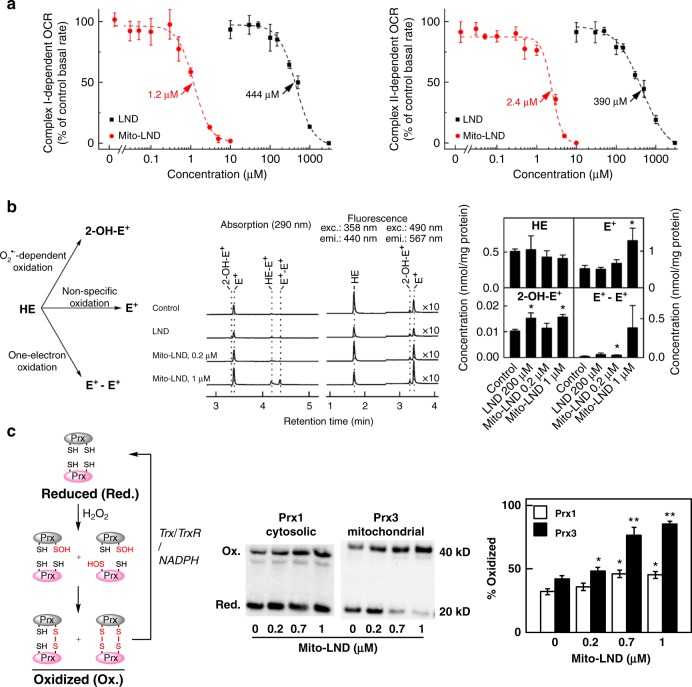
LND and Mito-LND impact the activity of mitochondrial complexes, ROS generation and peroxiredoxin oxidation. **a** Effect of LND and Mito-LND on the activity of mitochondrial complexes I and II. H2030BrM3 cells were pretreated with LND or Mito-LND for 24 h. The mitochondrial complex I and II oxygen consumption rates (OCR) are plotted against concentration of LND or Mito-LND. Dashed lines represent the fitting curves used for determination of the IC_50_ values. **b** Effect of LND and Mito-LND on cellular ROS production, as measured by HPLC-based analyses of the oxidation of the hydroethidine (HE) probe. The scheme of HE oxidation is shown in the left panel, HPLC fluorescence traces recorded are shown in the middle panel and HPLC quantitative data in the right panel. **c** Effect of LND and Mito-LND on redox status of cytosolic (Prx1) and mitochondrial (Prx3) peroxiredoxins in A549 cells. Diagram depicting the various redox states of Prx1 and Prx3 is shown in the left panel. Representative immunoblots from cells treated with Mito-LND are shown in the middle panel, and the quantitative analyses of the redox status of Prx1 and Prx3 are shown in the right panels. Data are presented as the means ± SD. *t* test versus control: *p < 0.05, **p < 0.01

Previous studies have shown that inhibition of mitochondrial bioenergetics can be a key antiproliferative and cytotoxic mechanism for mitochondria-targeted cationic agents^18–20^. Therefore, we hypothesized that inhibition of mitochondrial complexes I and II is the predominant initial mechanism for the growth inhibitory effects of Mito-LND in lung cancer cells. Oxygen consumption rates (OCR) were measured as a readout of mitochondrial function. H2030BrM3 cells were pretreated with LND or Mito-LND for 24 h and the activity of mitochondrial complexes I and II was tested. The results show that Mito-LND targets complexes I and II much more potently than LND (IC_50_s of 1.2 and 2.4 µM for Mito-LND versus 444 and 390 µM for LND) (Fig. 2a). Thus, Mito-LND is 370- and 162-fold more potent than LND for complexes I and II, respectively. We also compared the immediate OCR changes in permeabilized H2030BrM3 cells in response to different concentrations of LND and Mito-LND (Supplementary Fig. 5); Mito-LND dose-dependently and rapidly decreased OCR at greater than 100-fold lower concentrations as compared with LND. This rapid inhibition could represent an important initiating mechanism.

### Mito-LND induces ROS and mitochondrial oxidative stress

One of the consequences of inhibiting mitochondrial complexes I and II is enhanced generation of oxidizing species in mitochondria^15^. We have previously shown that the cell-permeable redox probe, HE, can be used to monitor antimycin A-induced mitochondrial superoxide^26^. Using HE as a redox probe, we determined that Mito-LND (1 µM) and LND (200 µM) increased ROS generation in H2030BrM3 lung cancer cells (Fig. 2b). The level of superoxide (O_2_^•–^)-specific product, 2-OH-E^+^, increased in Mito-LND and LND treated cells (Fig. 2b). Mito-LND also induced the formation of diethidium (E^+^-E^+^), an indicator of a potent, one-electron oxidant formation in mitochondria. From these results, we conclude that Mito-LND potently induces mitochondrial ROS generation in H2030BrM3 lung cancer cells.

Next, we investigated the oxidation of peroxiredoxins, which are endogenous intracellular sensors of H_2_O_2_ generation in mitochondria (Prx3) and in the cytosolic compartment (Prx1)^27–29^. Although low-molecular-weight thiols (cysteine and glutathione) and redox-regulated proteins with low p*K*_a_ cysteines react with H_2_O_2_ rather slowly (*k* = 1–10 M^−1^ s^−1^), protein thiol peroxidases such as peroxiredoxins and glutathione peroxidases react rapidly with H_2_O_2_ (*k* = 10^5^–10^8^ M^−1^ s^−1^)^28^. Mito-LND treatment of A549 (Fig. 2c) and H2030BrM3 (Supplementary Fig. 6) cells caused pronounced oxidation of mitochondrial Prx3 but had only minimal effecs on cytosolic Prx1. Because Prx3 accounts for nearly 90% of total mitochondrial peroxidase activity^27^, Mito-LND-induced generation of H_2_O_2_ likely overwhelmed the mitochondrial capacity to degrade peroxides.

### Mito-LND alters the AKT/mTOR signaling pathway

**Fig. 3:**
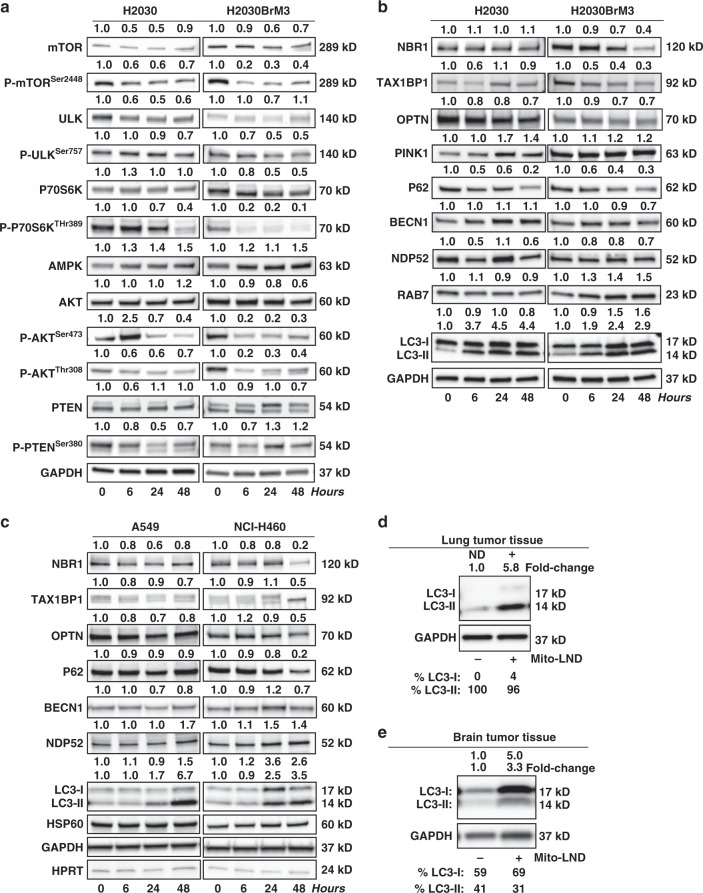
Mito-LND modulates energetic and autophagy signaling proteins in vitro and in vivo. **a** Mito-LND (2 µM) modulates AMPK and AKT/mTOR signaling cascades in H2030 and H2030BrM3 lung cancer cell lines over time. **b** Mito-LND (2 µM) modulates autophagy and specific mitophagy-linked proteins in H2030 and H2030BrM3 lung cancer cells. **c** Mito-LND (2 µM) modulates autophagy and specific mitophagy-linked proteins in A549 and NCI-H460 lung cancer cells. **d** Mito-LND induces autophagy in lung tumor tissues in mouse orthotopic model of lung cancer. **e** Induction of autophagy by Mito-LND in brain tumor tissues in the mouse brain lung metastasis model. The relative band intensities as determined by densitometry are indicated above each blot

AKT is a pro-survival factor that is constitutively activated (phosphorylated) in many cancers^30^. As shown in Fig. 3a, Mito-LND alters AKT/mammalian target of rapamycin (mTOR) signaling molecules. These are autophagy-linked energy-sensing proteins^31^. Specifically, Mito-LND decreased the levels of phosphorylated AKT (Fig. 3a). Mito-LND also decreased the phosphorylation of P70S6K (Fig. 3a) and other energy-sensing proteins in both the parental and metastatic lung cancer cell lines, indicating that Mito-LND specifically downregulates mTOR signaling. These results support the role of ULK (unc-51 like autophagy activating kinase) modifications in the downregulation of the AKT/mTOR signaling (Fig. 3a).

### Mito-LND induces autophagic cell death

We investigated if Mito-LND induces autophagy that promotes lung cancer cell death and inhibits lung cancer progression and metastases. Mito-LND treatment induced cytotoxic autophagy (Supplementary Fig. 7E) in lung cancer cells as evidenced by the accumulation of vacuoles and reduced cell viability (Supplementary Fig. 7A–D). Mito-LND treatment of H2030 and H2030BrM3 cells significantly inhibited cell viability over time (24, 48, and 72 h treatment). The LD_50_ for cell death induction was between 1 and 1.5 µM for H2030 and H2030BrM3 cells (Supplementary Fig. 7A–D). Mito-LND treatment did not induce pro-survival mechanisms in these cells at any concentration. Moreover, cells treated at or above the LD_50_ levels did not recover after Mito-LND was removed and the cells were replenished with fresh media and followed for an additional 48 h (Supplementary Fig. 7B5-8 and D5-8). To further investigate potential mechanisms underlying Mito-LND’s induction of cancer cell death, we analyzed lysates of cells treated with Mito-LND (2 µM) for proteins involved in energetics, apoptosis, and autophagy, including selective mitophagy markers. The results show that Mito-LND modulates key autophagy proteins, including specific mitophagy receptors, adaptor proteins, and energy-sensing molecules in both H2030 and H2030BrM3 cells. LC3, microtubule-associated protein light chain 3, is critical for cargo recruitment. The LC3 precursor is modified via a ubiquitination-like system generating soluble LC3-I, which is further modified upon autophagy induction to LC3-II, a membrane-bound phospholipid conjugate. LC3-II integrates into the autophagosome membrane and is considered a reliable early marker of autophagy induction. Mito-LND treatment increased levels of LC3-II from 1.9- to 4.4-fold in H2030 and H2030BrM3 cell lines, with sustained increases through 48 h (Fig. 3b). The LC3-interacting region (LIR) serves as a mechanistic basis for selective autophagy, including mitophagy, which specifically targets the degradation of damaged mitochondria via engagement of adaptor proteins (P62, NBR1, OPTN, BNIP3L)^32,33^. We evaluated the effects of Mito-LND on ubiquitin-binding mitophagy receptors (P62, NDP52, NBR1, OPTN), which also indicate autophagic flux or completion of the autophagic degradative process versus potential impairment of autophagosome turnover^33^. Mito-LND treatment of lung cancer cell lines decreased P62, which has a dual role in mitophagy, both in clustering of mitochondria as well as in downstream degradation. Mito-LND treatment of H2030 and H2030BrM3 cell lines resulted in decreased levels of additional mitophagy receptors including OPTN, TAX1BP1 and NDP52. However, Mito-LNDs impact on reduced TAX1BP1 and NDP52 appeared bimodal in H2030 cells, potentially due to the multiple autophagy-linked roles of these receptors, spanning from cargo recognition to autophagy biogenesis and autophagosome maturation.

PINK1 was included as an indicator of PINK1-Parkin-dependent mitophagy^34^. While basal levels of PINK1 are normally low, increases result from mitochondrial depolarization, damage, and ATP alterations, and such increases were noted in both cell lines treated with Mito-LND (Fig. 3b) with greater magnitude effects noted in H2030 cells. Beclin-1 (BECN1) is an autophagy regulator and potential tumor suppressor in multiple cancers, including NSCLC^35^. However, Mito-LND did not affect Beclin-1 levels in H2030 or H2030BrM3 cells (Fig. 3b). Thus, autophagy induction in these lung-derived cancer cell lines appears to be BECN1-independent. H2030BrM3 cells responded uniquely to Mito-LND with increased levels of RAB7 (Fig. 3b), a marker of late autophagy possibly linked to sustained ROS production^36^. Additional Ras mutant lung cancer cell lines, A549 and NCI-H460, responded similarly following Mito-LND treatment (Fig. 3c).

**Fig. 4:**
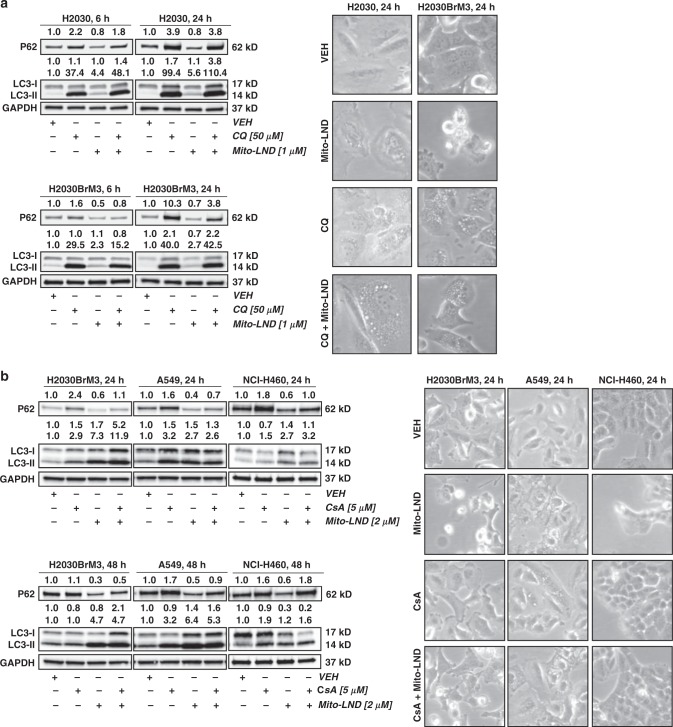
CQ and CsA block Mito-LND-induced autophagic flux and mitophagy. Mito-LND modulates autophagy and specific mitophagy-linked proteins in lung cancer cells as indicated. **a** Chloroquine (CQ, 50 µM) blocks late autophagy by inhibiting lysosomal acidification and inhibiting P62 degradation resulting in accumulation of LC3-II and autophagic vacuoles compared to vehicle (VEH) treated cells and mitigation of Mito-LND (1 µM) induced autophagy; (**a**, right) photomicrographs (200×). **b** Cyclosporin (CsA, 5 µM) blocks mitophagy and inhibits P62 degradation resulting in accumulation of LC3-II compared to vehicle treated cells and mitigation of Mito-LND (2 µM) induced mitophagy; (**b**, right) photomicrographs (200×)

Supplementary Figure 7 provides further support that Mito-LND induces complete autophagy in lung cancer cells. Vehicle-treated H2030 cells did not form autophagic vacuoles as illustrated by bright-field photomicrographs and were negative for monodansylcadaverine staining. In contrast, Mito-LND-treated cells showed markedly increased vacuolization characteristic of autophagy. Pretreatment with chloroquine (CQ), an inhibitor of late autophagy, and cyclosporin A (CsA), a mitophagy inhibitor, blocked Mito-LND-induced flux leading to an accumulation of vacuoles, LC3-II, and P62 levels (Fig. 4). The Mito-LND-induced autophagy was also observed in the tumor tissues collected from mice treated with Mito-LND (Fig. 3d, e), confirming that the mechanism established in vitro also occurs in vivo in both the mouse orthotopic model of lung cancer (5.8-fold induction) and the lung metastatic brain model (3.3-fold induction).

Other results also indicate that cell death induced by Mito-LND is mainly via autophagy rather than caspase-dependent apoptosis. While Mito-LND treatment resulted in increased levels of PARP and total cytochrome *c*, it did not induce caspase cleavage by itself (Supplementary Fig. 8) or in combination with chloroquine (Supplementary Fig. 9). Modest increases in cleaved CASP7 were noted following Mito-LND treatment of parental H2030 cells but not H2030BrM3 cells (Supplementary Fig. 9). In addition, Mito-LND increased the levels of the pro-death protein BAX and decreased the levels of BCL-2, consistent with autophagy-induced cell death (Supplementary Fig. 8)^37^. We also conducted flow cytometric analysis to determine the percentage of live, early apoptotic, late apoptotic and dead H2030BrM3 cells following Mito-LND treatment alone and in combination with autophagy blockers (chloroquine and cyclosporin A) (Supplementary Fig. 10). These data clearly indicate that Mito-LND does not induce apoptosis and that co-treatment with autophagy blockers significantly increases the percentage of live cells and decreases the percentage of dead cells (Supplementary Fig. 10A-B, D-E). Cyclosporin A pretreatment also reduces conversion of LC3-I to autophagic LC3-II in Mito-LND treated H2030BrM3 cells, supporting Mito-LND induced mitophagy as a key cancer cell death pathway (Supplementary Fig. 10C).

### Mito-LND inhibits orthotopic lung tumors in vivo

**Fig. 5:**
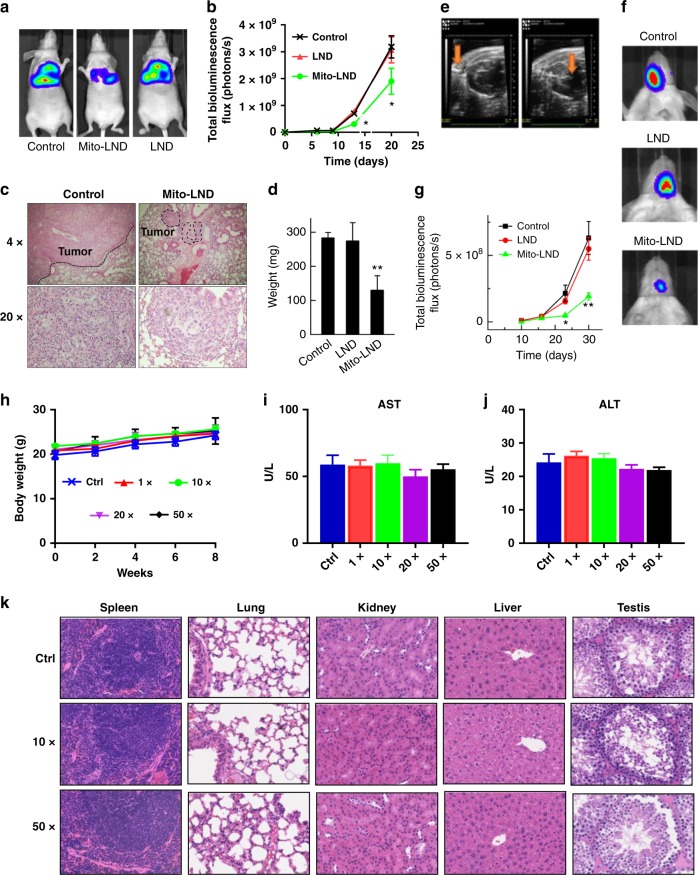
In vivo effects of LND or Mito-LND on lung tumor growth, brain metastasis and toxicity. **a** Representative bioluminescence images from mice bearing orthotopic H2030BrM3 lung tumors treated with vehicle, LND, or Mito-LND for 3 weeks. **b** Quantitative data for the bioluminescence imaging of lung tumors in control, LND- and Mito-LND-treated mice. **c** H&E staining orthotopic lung tumors from vehicle control or Mito-LND-treated mice (magnification: 4× and 20×). **d** The weight of the mediastinal lymph nodes harvested from control, LND- and Mito-LND-treated mice. **e** High-resolution echocardiography to visualize the position of the needle during intracardiac injection of lung cancer cells into the circulation. **f** Representative bioluminescence images of brain metastasis in control, LND, and Mito-LND-treated mice. **g** Quantitative data for the bioluminescence imaging of brain metastases. **h** Measured body weight. **i**, **j** AST and ALT blood levels in mice after 8 weeks of treatment with Mito-LND. The 1×, 10×, 20×, and 50× doses are relative to the effective dose (1× = 7.5 µmol/kg). **k** Histological images of key organs collected from mice treated with or without Mito-LND. Data are presented as the means ± SEM, *n* = 5 or 6, *t* test versus control: *p < 0.05, **p < 0.01

We conducted a study using an orthotopic model of lung adenocarcinoma in nude mice. H2030BrM3 cells (10^6^ cells/50 µg of growth factor-reduced Matrigel in 50 µL of RPMI-1640) were injected into the lung. One week after injection, mice were treated with the same dose of LND or Mito-LND (7.5 µmol/kg) or vehicle (corn oil) by oral gavage 5 days/week for 3 weeks. Equimolar doses were administered to illustrate the markedly enhanced potency of Mito-LND relative to LND. LND was not effective, as expected, as the applied dose (7.5 µmol/kg) is below its levels typically used for xenograft studies. In contrast, even at this relatively low dose, Mito-LND significantly decreased tumor progression (>40% inhibition of the BLI signal intensity; Fig. 5a–c) and lymph node metastasis (>50% inhibition of the weight of the mediastinal lymph nodes; Fig. 5d), demonstrating Mito-LND’s markedly enhanced potency against both lung cancer progression and metastasis. To test if these effects are limited to the H2030BrM3 orthotopic mouse model, we carried out similar experiments in vivo using A549 cells. As shown in Supplementary Fig. 11A, B, Mito-LND decreased the rate of growth of A549 tumor xenografts, whereas LND did not inhibit tumor growth.

### Mito-LND inhibits lung cancer brain metastasis in vivo

We assessed the effects of Mito-LND versus LND in a Nod/Scid mouse model of brain metastasis in which luciferase-expressing H2030BrBM3 cells were injected via intracardiac insertion into the arterial circulation. To ensure the accuracy of injection and precision in the quantity of the cells injected, we used high-resolution echocardiography to guide the needle into the left ventricle. In Fig. 5e, the needle’s position is discernible. Mice were imaged for firefly luciferase expression in the tumor cells at multiple time points post-injection. Quantitative bioluminescence imaging data show a marked decrease in lung cancer brain metastasis in Mito-LND-treated mice but not in those treated with LND (Fig. 5f, g). Similar effects of Mito-LND were also observed in the A549 brain metastasis mouse model (Supplementary Fig. 11C, D).

### Maximum tolerated dose and safety of Mito-LND

We conducted an 8-week toxicology study of Mito-LND in mice, including a Modified Irwin Screen (a comprehensive observational battery to screen for central nervous system effects)^38^. Modifications of this test have been used to identify changes in neurological function and potential neurotoxicity^39^. The screen included 35 distinct measures of sensorimotor, neurological, and autonomic nervous system function. Considering the effective dose (ED) of Mito-LND is 7.5 µmol/kg, we did not observe any significant differences in body weight (or organ weights) between control and Mito-LND-treated mice at 10 × (75 µmol/kg), 20 × (150 µmol/kg), and 50 × (375 µmol/kg) the ED (Fig. 5h), nor did we notice any changes in the 35 metrics of the Modified Irwin Screen at any dose (Supplementary Table 1). No adverse effects were seen in tissues by histopathology or liver function indicators (AST, ALT) (Fig. 5i–k). Thus, Mito-LND, even at doses up to 50 times the ED, administered for 8 weeks, was well tolerated and did not show any observable toxicity.

### Mito-LND is selective for cancer cells and tumor tissue

**Fig. 6:**
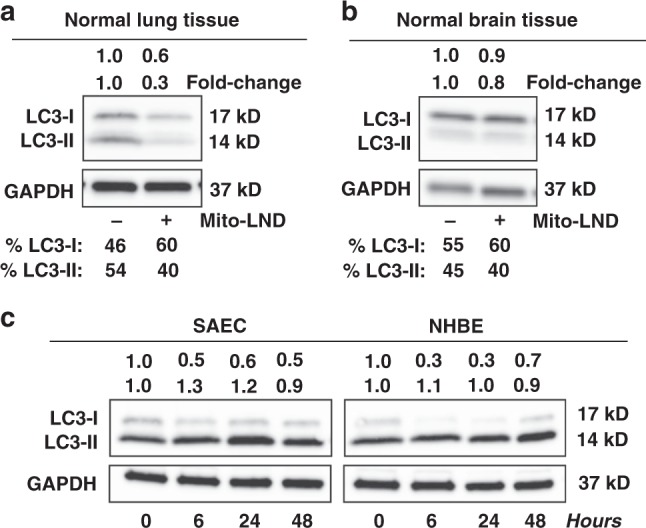
Mito-LND does not induce autophagy in normal lung and brain tissue or normal lung cell lines. **a** Mito-LND treatment in vivo does not induce autophagy in normal lung tissue compared to the 5.8-fold increase of the autophagic form of LC3-II in adjacent lung tumor tissue (see Fig. 3d) in the mouse orthotopic model of lung cancer. **b** Mito-LND treatment in vivo does not induce autophagy in normal brain tissue compared to the 3.3-fold increase of autophagic form of LC3-II in adjacent brain tumor tissue (see Fig. 3d) in the mouse brain lung metastasis model. **c** Effects of Mito-LND (2 µM) on proteins related to signaling and autophagy in normal lung cells. Mito-LND shows a minor impact on autophagy induction in normal cell lines (SAEC and NHBE) compared to cancer cell lines (see Fig. 3b, c)

As stated above, Mito-LND was non-toxic in vivo (Fig. 5h–k). Moreover, Mito-LND treatment did not induce autophagy in normal mouse lung and brain tissues (Fig. 6a, b). In sharp contrast, as described above, Mito-LND induced autophagy in both mouse lung and metastatic brain tumor tissues (Fig. 3d, e). Additional in vitro experiments were conducted to assess Mito-LND’s selectivity towards cancer cells compared to non-tumorigenic small airway epithelial cells (SAEC) or normal human bronchial epithelial (NHBE) cells. As shown in Supplementary Fig. 7F, Mito-LND selectively induced cell death in tumorigenic H2030BrM3 cells, but not in non-tumorigenic SEAC cells. In contrast to inducing autophagy in RAS mutant lung cancer cell lines (Figs. 3 and 4), Mito-LND failed to induce autophagy in non-tumorigenic SAEC and NHBE cell lines (Fig. 6c). These data support that Mito-LND’s induction of autophagic cell death, mediated through mitophagy, occurs specifically in cancer cell lines and tumor tissue, without affecting normal cell lines or normal surrounding tissue.

## Discussion

Mito-LND has distinctly different effects than LND and the effects of LND on energy metabolism have been an active area of research^11^, and targeting mitochondrial bioenergetics is emerging as an effective approach for cancer treatment and prevention. Although tumor cells rely mostly on aerobic glycolysis to generate ATP (the Warburg effect), many studies have shown that mitochondria are indeed functional in most tumor cells and play a key role in regulating tumor growth and metastasis^40,41^. Studies have suggested that LND’s effects on tumor cells may predominantly occur by inhibiting glycolysis, specifically hexokinases that phosphorylate glucose to glucose 6-phosphate (specifically mitochondria membrane-associated hexokinase II)^42^. Our results, however, indicate that at the low µM Mito-LND concentrations that effectively inhibit tumor growth, Mito-LND is not likely to directly inhibit hexokinase II activity (Supplementary Fig. 12); significant inhibition of hexokinase is only observed with 100 µM Mito-LND. However, it remains to be established if the accumulation of Mito-LND in cell mitochondria could lead to inhibition of mitochondria-localized hexokinase II in intact cells. LND can also prevent the efflux of lactate from cells through inhibition of monocarboxylic acid transporter^43^, although this is unlikely to be true for Mito-LND due to its lack of a free carboxyl group (Fig. 1a) and the lack of significant hydrolysis of the Mito-LND ester bond in intact cells (Supplementary Fig. 4). Recently, 100–200 µM LND was shown to inhibit mitochondrial complex II in the respiratory chain of isolated mitochondria^12^. The central focus of the work we report herein was to markedly enhance the effects on mitochondrial bioenergetics, and thus inhibit cancer cell growth and metastasis, by creating a mitochondrial-targeted LND.

**Fig. 7:**
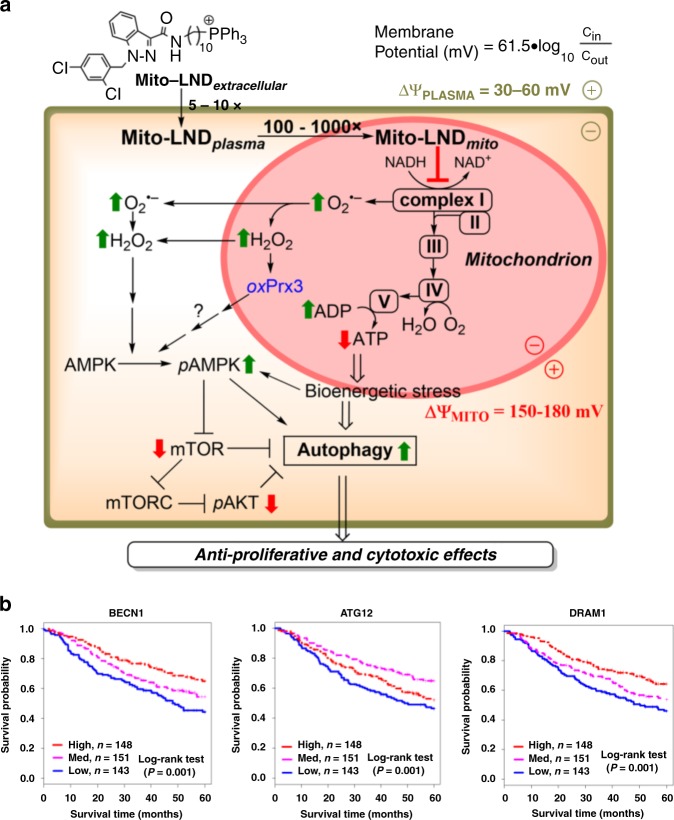
Targeting bioenergetic function and redox signaling by Mito-LND. **a** Mito-LND inhibits mitochondrial complexes I and II, leading to the depletion of cellular ATP and stimulation of ROS; activation of autophagy, leading to inhibition of cell proliferation, and invasion. TPP^+^-linked compounds accumulate to high levels in tumor mitochondria according to the Nernst equation. **b** Kaplan–Meier survival curves. Analysis of a Thoracic Surgery mRNA expression database (containing 440 lung adenocarcinoma cases) shows that high expression of pro-autophagic markers is significantly associated with increased patient survival

Increased targeting of LND to tumor cells has become an intense area of research. The efficacy of LND can be enhanced using targeted delivery systems such as nanoparticles. For example, the tumor-suppressive effects of LND were markedly improved using EGFR receptor-targeting nanoparticles^44^. Such nanoparticle carriers can enhance the antitumor efficacy of LND by about 100-fold. We found that conjugating a TPP^+^ moiety to LND via an alkyl side chain vastly increased the potency for inhibiting lung cancer cell proliferation. The IC_50_ of Mito-LND is 188- to 300-fold lower than the IC_50_ of LND for inhibiting the proliferation of lung cancer cells (Fig. 1b). Furthermore, Mito-LND inhibits both complexes I and II with IC_50_s of 1.2 and 2.4 µM, respectively, compared with LND’s effects on complex I and II that occur at concentrations in the range of 400 µM (Fig. 2a). Mito-LND is a positively charged molecule that is more selectively sequestered in tumor mitochondria (as compared with mitochondria of normal cells) because of the increased negative plasma membrane and mitochondrial transmembrane potentials in cancer cells (Fig. 7a)^25^. Mito-LND’s inhibition of complexes I and II occurs within minutes of exposure, which suggests that this inhibition is a key initiating event for its effects on tumor cells.

The inhibition of mitochondrial complexes I and II is known to enhance the generation of oxidizing species in mitochondria^15^. Consistent with this premise, Mito-LND markedly stimulated ROS generation in tumor cells (Fig. 2b). As described in a recent review on mitochondrial oxidants^26^, HE undergoes one-electron oxidation in mitochondria of cancer cells treated with mitochondria-targeted agents. The oxidant, higher oxidation peroxidase or hydroxyl radical/iron-oxo species, is presumably formed from the interaction between heme proteins or redox active iron and H_2_O_2_ generated from the inhibition of mitochondrial complexes. The intermediate radical (HE radical cation) derived from HE reacts rapidly with O_2_^•–^ to form a diagnostic product, 2-OH-E^+^_,_ and/or other oxidation products, E^+^ and E^+^–E^+^. HE-derived dimers are characteristic marker products of the probe oxidized by a peroxidatic mechanism^45^. Compared with LND, Mito-LND is significantly more potent in inducing dimer formation from the oxidation of HE in mitochondria (Fig. 2b). Although Mito-SOX (mitochondria-targeted HE) has been used in some studies to indicate mitochondrial O_2_^•–^ formation, recent interpretations implicate a nonspecific oxidation mechanism (catalyzed by peroxidatic activity) as the likely pathway and indicate that red fluorescence derived from Mito-SOX results predominantly from the two-electron oxidation product and not from the O_2_^•–^-specific hydroxylated product. Clearly, the hydroxylated product from Mito-SOX (isolated in low yield due to difficulty in the extraction) must be characterized to implicate mitochondrial O_2_^•–^. The cell-permeable HE is therefore a more suitable probe that can be used to specifically determine total cellular (cytosolic and mitochondrial) O_2_^•–^ (Fig. 2b). Since superoxide dismutase can rapidly generate H_2_O_2_ from O_2_^•–^, we used the redox state of peroxiredoxins as endogenous intracellular sensors of excess H_2_O_2_ generation in mitochondria (Prx3) and the cytosolic compartment (Prx1). Mito-LND treatment caused pronounced oxidation of mitochondrial Prx3 but had only minimal effects on cytosolic Prx1 (Fig. 2c), which implies that the ROS generation in Mito-LND-treated cells was predominantly localized in the mitochondria. Relative to Mito-LND, 200-fold higher doses of LND were required to induce ROS generation in lung cancer cells, and LND did not induce Prx3 oxidation in cells treated with doses equivalent to those of Mito-LND.

While inhibition of mitochondrial complexes I and II, and the resulting generation of ROS, are early events associated with Mito-LND, we propose that autophagic cell death is a major pathway by which Mito-LND ultimately exerts antiproliferative and cytotoxic effects. Autophagy is a universal cellular process by which cytoplasmic components are directed to the lysosome for degradation. Macroautophagy, microautophagy, and chaperone-mediated autophagy are the three main types of autophagy, although additional specialized forms of autophagy have been mechanistically defined^46^. Recently, autophagy and mitophagy (which involves selective degradation and removal of mitochondria via the macroautophagic pathway) are emerging as important mechanisms by which certain anticancer agents induce cell death, especially as a consequence of cellular stress. It was previously reported that mitochondria-targeted Mito-Q predominantly promotes autophagic cell death (with apoptosis as a minor pathway) in breast cancer cells but not in normal cells via mechanisms involving ROS generation^21^. The role of autophagy in cancer is complex, with two major and seemingly opposing functions^47^. Autophagy can be either tumor suppressive or oncogenic depending on the cancer type and cellular environment^48^. With nutrient deprivation, autophagy induction can promote cell survival. Consequently, a large body of research has focused on utilizing autophagy inhibitors to target cytoprotective autophagy, a form which sustains cancer cell survival and confers resistance to therapy^49^. However, in response to other environmental stressors including ROS generation, autophagy induction can lead to cancer cell death via autophagic cell death^50^. Based on our results, we propose that Mito-LND decreases cell viability and induces autophagic cell death in H2030, H2030BrM3, A549, and NCI-H460 lung cancer cells.

The role of autophagy in human lung cancer progression, metastasis, and prognosis is still an emerging area of research. In a study of >100 patients, high BECN1 expression correlated with well-differentiated NSCLC and reduced lymph node metastasis. The same study reported that low BECN1 expression coupled with high P62 expression is significantly associated with shorter patient survival time^51^. Similarly, an mRNA expression database (containing 440 lung adenocarcinoma cases) for pro-autophagy markers was queried and the results showed that high expression of BECN1, ATG12, and DRAM1 are closely linked to increased survival among lung adenocarcinoma patients (Fig. 7b).

In this study, we show that modification of an antiglycolytic drug, LND, to a mitochondria-targeted drug, Mito-LND, potently inhibits lung cancer growth and metastasis. Through its inhibition of mitochondrial bioenergetics, Mito-LND induces ROS in lung cancer cells, which results in the oxidation of mitochondrial peroxiredoxin, inactivation of the AKT/mTOR/p70S6K redox signaling axis and autophagic cell death, as summarized in Fig. 7a. Mito-LND is significantly different from other mitochondria-targeted agents in a number of ways: it is the least toxic of all mitochondria-targeted compounds that we have tested in mice so far, it induces autophagic death at low micromolar concentrations that are readily achieved in vivo, and it markedly inhibits both primary lung tumors and their brain metastases. These findings reveal that inhibiting mitochondrial complexes that ultimately result in autophagic cell death is a key pathway through which mitochondria-targeted compounds inhibit proliferation, invasion, and metastasis of lung cancer cells. We have previously shown that mitochondria-targeted agents protect against the cardiotoxicity and nephrotoxicity of standard-of-care chemotherapeutics in animal models^25,52–54^. Our results with Mito-LND (Supplementary Fig. 13) suggest that combining mitochondria-targeted anticancer agents such as Mito-LND with platinum-based chemotherapeutics may result in synergistic inhibition of proliferation of lung cancer cells. Overall, the obtained results represent a potentially significant autophagy-related therapeutic approach for mitigating lung cancer development and brain metastases.

## Methods

### Synthesis and characterization of Mito-LND and Alkyl-LND

Mito-LND and Alkyl-LND were synthesized according to the reaction scheme shown in Supplementary Fig. 14 and characterized by nuclear magnetic resonance (NMR) and mass spectrometry. Experimental details of the synthesis are provided in Supplementary Methods. Supplementary Figure 15 shows the phosphorus (^31^P), proton (^1^H) and carbon (^13^C) NMR spectra of synthesized Mito-LND. Supplementary Figure 16 shows the proton (^1^H) and carbon (^13^C) NMR spectra of synthesized Alkyl-LND.

### Cell lines

H2030 lung adenocarcinoma cells, which have a *KRAS*^G12C^ mutation^55^, were purchased from ATCC (catalog #CRL-5914). A549 (ATCC #CCL-185) is a human adenocarcinoma alveolar basal epithelial cell line that has a *KRAS*^G12S^ mutation. NCI-H460 is a human epithelial cell line derived from large cell lung carcinoma tissue (ATCC catalog #HTB-177) and has a *KRAS*^Q61H^ mutation. H2030BrM3 cells (isolated from brain metastases of H2030 cells) were generously provided by Dr. Joan Massagué (Cancer Biology and Genetics Program, Memorial Sloan Kettering Cancer Center, New York, NY). Normal lung cell lines, SAEC and NHBE, were obtained from Lonza (catalog #CC-2540 and #CC-2547, respectively). The H2030BrM3 cells consistently form brain metastases in 100% of animals compared to ~10% efficiency of their parental cell line^56^. These cell lines were engineered to express a green fluorescent protein (GFP)-luciferase fusion protein, which enabled monitoring of in vivo tumor growth and metastasis. All cells were kept frozen in liquid nitrogen and were used within 20 passages after thawing. H2030 and H2030BrM3 lung cancer cells were cultured under standard conditions (37 °C and 5% CO_2_) in RPMI medium (Thermo Fisher Scientific, #11875) supplemented with 10% fetal bovine serum, 100 units/mL penicillin, and 100 µg/mL streptomycin (Thermo Fisher Scientific, Waltham, MA). A549 and NCI-H460 lung cancer cells were cultured in F-12K medium (Thermo Fisher Scientific, #21127022) and ATCC-formulated RPMI 1640 (Thermo Fisher Scientific #A1049101), respectively, with supplements listed above. The normal lung cell line SAEC was cultured in SAGM small airway epithelial cell growth medium (Lonza #CC-3118) and NHBE cells were cultured in BEGM bronchial epithelial cell growth medium (Lonza # CC-3170). All cell lines used in this study were authenticated on a regular basis and verified to be free of *Mycoplasma* contamination (Universal Mycoplasma Detection Kit, ATCC).

### ROS probe and standards of oxidation products

Hydroethidine (HE) was purchased from Invitrogen (Carlsbad, CA). A stock solution of HE (20 mM) was prepared in deoxygenated DMSO and stored at −80 °C until use. Ethidium cation (bromide salt) was purchased from Sigma-Aldrich (St. Louis, MO). The hydroxylated oxidation product from HE (2-hydroxyethidium, 2-OH-E^+^) was prepared by reacting the probe with Fremy’s salt^45,57^. The dimeric product (E^+^–E^+^) was prepared by reacting the probe with excess potassium ferricyanide^57^. Synthesized standards of all oxidation products of HE were purified by high-performance liquid chromatography (HPLC).

### HPLC analyses

HPLC-based measurements of HE and its oxidation products were performed using an Agilent 1100 HPLC system (Santa Clara, CA) equipped with absorption and fluorescence detectors and a refrigerated autosampler (4 °C). The samples (50 µL) were injected into a reverse phase column (Phenomenex, Kinetex C_18_, 100 mm × 4.6 mm, 2.6 µm) equilibrated with 20% acetonitrile (MeCN), 80% water containing 0.1% trifluoroacetic acid. The compounds were eluted by increasing the content of MeCN from 20 to 56% over 4.5 min at a flow rate of 1.5 mL/min. The detection parameters were as previously reported^26,57^.

### Monodansylcadaverine staining of autophagic vacuoles

H2030 (7.5 × 10^3^ cells/chamber) and H2030BrM3 (1.2 × 10^5^ cells/chamber) were plated in eight-well glass chamber slides (Thermo Fisher Scientific) with RPMI complete medium, allowed to adhere for at least 24 h, and then treated with 2 µM Mito-LND or DMSO (<0.01%) dissolved in complete RPMI medium for 4 h. The growth medium was removed, and cells were stained with monodansylcadaverine (MDC; Sigma-Aldrich) for 30 min at 37 °C to label acidic autophagic vacuoles. Cells were washed three times with phosphate-buffered saline (Thermo Fisher Scientific), and MDC fluorescence was visualized using the Cytation 5 Cell Imaging Multi-Mode Reader (BioTek, Winooski, VT) and the Eclipse Ts2 inverted fluorescent microscope (Nikon, Melville, NY) at 20× magnification with excitation/emission wavelengths of 460/535 nm.

### Lysate collections and Western blot analyses

H2030, H2030BrM3, A549, NCI-H460, SAEC (Human Small Airway Epithelial Cells) and NHBE (Normal Human Bronchial Epithelial Cells) cells were seeded in T-25 flasks and adhered overnight prior to treatment with 2 µM Mito-LND or DMSO dissolved in cell line specific medium. To assess the impact of pharmacologically blocking autophagy, lung cancer cells were pretreated with 50 µM chloroquine diphosphate (Sigma-Aldrich) or vehicle (water) for 2 h prior to the addition of 1 µM Mito-LND or vehicle (DMSO). To specifically evaluate autophagy blockade via mitophagy inhibition, lung cancer cells were pretreated with 5 µM cyclosporin A (CsA; Sigma-Aldrich) or vehicle (DMSO) for 2 h prior to the addition of 2 µM Mito-LND or vehicle (DMSO). Brightfield photomicrographs were obtained prior to lysate collection using an Olympus CK2 inverted microscope at 200× magnification. Cell lysates were prepared from cells harvested at 0, 6, 24, and 48 h post-treatment using lysis buffer (1% Triton X-100, 50 mM HEPES, pH 7.4, 150 mM NaCl, 1.5 mM MgCl_2_, 1 mM EGTA, 100 mM NaF, 10 mM sodium pyrophosphate, 1 mM sodium orthovanadate, 10% glycerol) with complete EDTA-free protease and PhosSTOP phosphatase inhibitors (Sigma-Aldrich). Protein was quantified using the DC protein assay (Bio-Rad, Hercules, CA). Approximately 20 µg of protein was loaded in precast 4–20% Mini-Protean TGX gels (Bio-Rad), run for 1 h, transferred to a PVDF membrane with the Trans-Blot® Turbo™ system (Bio-Rad) for 30 min, blocked for 1 h at room temperature, incubated overnight with primary antibodies, and incubated with the secondary antibody for 1 h. Images were captured via the ChemiDoc Molecular Imager and bands were quantified with ImageLab analysis software (both from Bio-Rad). Expression levels were determined by chemiluminescent immunodetection and normalized to appropriate loading controls. Uncropped and unprocessed Western blot images are presented in Supplementary Figs. 17–22. Immunoblotting was performed using commercially available antibodies from Abcam (Cambridge, MA): NDP52 (#ab68588; 1:500); BD Biosciences (San Jose, CA): P62 (#5114; 1:1000); Cell Signaling Technology (Danvers, MA): AKT (#4691; 1:1000), AMPK (#2532; 1:1000), Bax (#5023; 1:1000), Bcl-2 (#2876; 1:1000), Beclin-1 (#3738; 1:750), Caspase 3 (#9665; 1:500), Caspase 7 (#12827; 1:1000), Caspase 9 (#9508; 1:1000), GAPDH (#2118; 1:40,000), LC3 (#4108; 1:1000), mTOR (#2983; 1:1000), NBR1 (#9891; 1:500), PARP (#9532; 1:500), P70 S6 Kinase (#2708; 1:1000), PTEN (#9552; 1:1000), phospho-AKT^Ser473^ (#4060; 1:1000), phospho-AKT^Thr308^ (#13038; 1:1000), phospho-mTOR^Ser2448^ (#5536; 1:500), phospho-P70 S6 Kinase^Thr389^ (#9234; 1:1000), phospho-PTEN^Ser380^ (#9551; 1:1000), phospho-ULK^Ser757^ (#6888; 1:1000), RAB7 (#9367; 1:1000), TAX1BP1 (#5105; 1:1000), and ULK (#8054; 1:1000); Novus Biologicals (Littleton, CO): PINK1 (#BC100–494; 1:500); Proteintech Group, Inc. (Rosemont, IL): Optineurin (#10837–1-AP; 1:500); and Santa Cruz Biotechnology (Dallas, TX): Cytochrome *c* (#sc-13156; 1:500), HSP60 (#sc-13966; 1:5000).

### Flow cytometric analyses

H2030BrM3 (1 × 10^6^) were seeded in T-25 flasks (Corning, Thermo Fisher Scientific) and allowed to adhere for 24 h at 37 °C in a 5% CO_2_ atmosphere. Cells were washed and pretreated for 2 h with either vehicle, 50 µM chloroquine or 5 µM cyclosporin A (total volume: 5 mL). After 2 h, 1 mL of the final treatment was added directly to the flask such that the total treatment volume was 6 mL. The final treatments were (A) vehicle (0.01% DMSO), (B) 50 µM chloroquine, (C) 5 µM cyclosporin A, (D) 2 µM Mito-LND, (E) 50 µM chloroquine and 2 µM Mito-LND, or (F) 5 µM cyclosporin A and 2 µM Mito-LND. After 48 h, nonadherent cells were collected by centrifugation and adherent cells were harvested by trypsinization for apoptosis staining. Approximately 5 × 10^5^ cells were stained with Annexin V FITC (BD Biosciences) and propidium iodide (BD Biosciences) in 1× Annexin V binding buffer (BD Biosciences) following manufacturer’s protocols. Appropriate controls included no stain, Annexin V FITC only, and propidium iodide only. An example of gating thresholds is shown in Supplemental Fig. 23. All treatments were performed in triplicate. Flow cytometric analysis was performed on the Ze5™ cell analyzer (Bio-Rad) using the Everest software package. A minimum of 10^3^ cells were counted and data were analyzed using FlowJo software (FlowJo, LLC, Ashland, OR).

### Cellular viability, proliferation, and cytotoxicity assays

H2030 (3 × 10^3^ cells/well) and H2030BrM3 (1 × 10^4^ cells/well) were plated in black-walled 96-well plates, treated with Mito-LND (0.75–1.5 µM) or vehicle control (dimethyl sulfoxide [DMSO]; Sigma-Aldrich) and cell viability was determined at 24 h and 48 h post-treatment using the Live/Dead Viability/Cytotoxicity kit (Thermo Fisher Scientific) per the manufacturer’s instructions. Fluorescence imaging for the viability assay was conducted using the SpectraMax® MiniMax™ Imaging Cytometer (Molecular Devices, Sunnyvale, CA) with excitation/emission wavelengths of 460/535 nm. Cell proliferation was measured using a label-free, noninvasive cellular confluence assay (IncuCyte Live Cell Imaging Systems, IncuCyte FLR, Essen Bioscience, Ann Arbor, MI), as recommended by the manufacturer^20^. To determine the cytotoxicity of Mito-LND, we used the Sytox Green-based assay per the manufactures instructions^19^. Cells were treated for 24 h, and dead cells were monitored in real time in the presence of 200 nmol/L Sytox Green (Invitrogen) under an atmosphere of 5% CO_2_:95% air at 37 °C.

### Mitochondrial function in intact and permeabilized cells

The mitochondrial function in intact and permeabilized cells was monitored using a Seahorse XF96 Extracellular Flux Analyzer (Agilent, Santa Clara, CA)^14,22^. Activities of mitochondrial respiratory complexes in permeabilized cells were measured according to the manufacturer’s instructions. Briefly, intact cells were permeabilized using 1 nmol/L Plasma Membrane Permeabilizer (PMP, Agilent) immediately prior to measuring the OCR. The oxygen consumption derived from mitochondrial complexes was measured using different mitochondrial substrates (e.g., pyruvate/malate for complex I and succinate for complex II)^58^. Rotenone, malonate and antimycin A were used as specific inhibitors of mitochondrial complexes I, II, and III, respectively.

### Redox blotting

The redox status of cytosolic and mitochondrial peroxiredoxins (Prx1 and Prx3, respectively) was determined by redox Western blotting, adapted from Cox et al. ^26,27,29^. Briefly, after treatment, cells were washed quickly with HBSS, overlayed with a thiol blocking buffer containing 0.1 M *N*-ethylmaleimide (NEM), 50 mM NaCl, 40 mM HEPES, pH 7.4, 1 mM PMSF, 1 mM EDTA, 1 mM EGTA, and 10 µg/mL catalase. Cells were harvested and incubated at room temperature for 15 min, pelleted (5 min, 800 × *g*) and the pellet lysed in the small volume of the thiol blocking buffer supplemented with 1% CHAPS (3-((3-cholamidopropyl)dimethyl-ammonio)-1-propanesulfonate). Lysates were stored at −80^o^C until analysis. On the day of analysis, the lysates were thawed on ice and then centrifuged for 5 min (8000 × *g*, 4 °C). The supernatants were run on nonreducing SDS-PAGE. The blots were probed with anti-Prx1 (sc-7381; 1:500) and anti-Prx3 (sc-59661; 1:500) antibodies (both from Santa Cruz Biotechnology), followed by HRP-conjugated secondary antibodies. After chemiluminescence measurements were taken using a luminescence imager, the blots were stripped and probed for β-actin (indicator of protein load).

### Quantitation of intracellular LND and Mito-LND by LC/MS

Cells were grown in 10-cm dishes and incubated with the compounds for 24 h in complete medium. Liquid chromatography–mass spectrometry (LC-MS/MS) analyses were performed on cell extracts using Shimadzu Nexera2 UHPLC system equipped with UV-Vis absorption and triple quadrupole mass spectrometry (LC-MS8030) detectors. Detection of lonidamine was accomplished using Raptor Biphenyl column (Restek, 100 mm × 2.1 mm, 2.7 µm) equilibrated with mobile phase containing water:methanol (MeOH) mixture (4:1). LND was eluted by increasing the content of MeOH from 20 to 100% over 5 min and detected in the multiple reaction monitoring (MRM) mode using the transition of 319.10 > 274.95 (negative mode). Mito-LND was analyzed using a Kinetex F5 column (Phenomenex, 100 mm × 2.1 mm, 1.7 µm) equilibrated with a mobile phase containing water (75%), MeCN (25%) and formic acid (0.1%). Mito-LND was eluted by increasing the content of MeCN in the mobile phase from 25 to 100% over 5 min and detected in the MRM mode using the transition of 720.20 > 262.00 (positive mode). For both LND and Mito-LND analyses, the mobile phase flow rate was set at 0.5 mL/min.

### Uptake of Mito-LND into isolated mitochondria

For measuring uptake of Mito-LND into isolated mitochondria^59,60^, we incubated Mito-LND (10 µM) with mouse liver mitochondria (1.5 mg/mL) in nonionic mannitol plus a sucrose-based buffer (220 mM mannitol, 70 mM sucrose, 10 mM KH_2_PO_4_, 5 mM MgCl_2_, 2 mM HEPES, 1 mM EGTA, 0.2% fatty acid-free BSA, pH 7.2) at room temperature for 2 min, followed by additional 2 min incubation in the presence or absence of mitochondrial uncoupler, FCCP (1 µM). Next, the suspension was centrifuged (7000 *g*, 1 min) and the concentration of Mito-LND in the supernatant was measured by HPLC (Agilent 1100 HPLC system with UV-Vis and fluorescence detectors). The mitochondrial supernatant was injected onto a Raptor Biphenyl column (Restek, 50 mm × 4.6 mm, 2.7 µm) equilibrated with mobile phase containing water:acetonitrile mixture (1:1 by vol.) and trifluoroacetic acid (0.1% by vol.). Mito-LND was eluted by increasing the acetonitrile content in the mobile phase from 50 to 100% (by vol.) over 3 min, at a flow rate of 2 mL/min. For Mito-LND quantification, fluorescence traces (excitation at 300 nm, emission at 420 nm) were used.

### Transwell invasion assay

Boyden chamber transwells pre-coated with growth-factor-reduced Matrigel Matrix were purchased from Fisher Scientific (Pittsburgh, PA). Transwell invasion assays were performed according to the manufacturer’s protocol. Briefly, 2–3 × 10^5^ cells were seeded into each transwell, filled with serum-free culture medium. The bottom wells were filled with cell culture medium or Waymouth’s medium with 10% FBS and either LND or Mito-LND. Controls received an equivalent amount of DMSO. After 36 h, cells were fixed with 10% formalin and stained with 5% crystal violet in 70% ethanol. Invaded cells were counted at a magnification of 10 × in three randomly selected areas of each transwell, and the results were normalized to the control.

### Toxicity studies of Mito-LND

All procedures were in accordance with the Medical College of Wisconsin Institutional Animal Care and Use Committee. An 8-week subchronic toxicity study of Mito-LND was conducted in A/J mice (6 weeks old, from Jackson Laboratories). During treatment, a Modified Irwin Screen employing 35 distinct measurements was used to assess sensorimotor, neurological, and autonomic nervous system function. A/J mice were treated with vehicle control or different doses of Mito-LND, given via oral gavage 5 days per week for 8 weeks. Body weights were measured weekly. After 8 weeks of treatment, serum was collected to measure alanine transaminase (ALT) and aspartate aminotransferase (AST) using an Ortho Clinical Diagnostic Vitros Fusion 5.1 analyzer. Mice were euthanized by CO_2_ asphyxiation. Organs and tissues were subjected to pathological analysis. All tissues were fixed in a 10% zinc formalin solution overnight and stored in 70% ethanol for histopathology evaluation. Serial tissue sections (5 μm each) were made, stained with hematoxylin and eosin (H&E), and examined histologically under a light microscope.

In vivo lung cancer orthotopic model. We used an orthotopic model of lung adenocarcinoma cells (H2030BrM3 cells) in athymic nude mice to evaluate the inhibitory effect of Mito-LND on lung tumor growth. Nude mice (5 weeks) were anesthetized with isoflurane and placed in the right lateral decubitus position. A total of 1 × 10^6^ H2030BrM3 cells in 50 μg of growth factor reduced Matrigel in 50 μL of RPMI1640 medium were injected into the left lung through the left rib cage. One week after injection, mice were treated 5 days per week for 3 consecutive weeks with LND, Mito-LND or vehicle control. Tumor growth and metastases were monitored over time by bioluminescence (after injection of D-luciferin, 150 µg/g) using Lumina IVIS 100 imager (Perkin Elmer, Waltham, MA). Mice were euthanized at day 28; lung tissues in each group were weighed.

Brain metastases mouse model. Female NOD/SCID mice (age 5 weeks) were used for the brain metastases studies. H2030BrM3 cells (2 × 10^5^) were suspended in PBS (0.1 mL) and injected into the left ventricle under ultrasound guidance (ECHO 707, GE, Milwaukee, WI). One day after engrafting lung cancer cells in the arterial circulation, mice were randomly grouped into treatment groups: vehicle control, LND, or Mito-LND. Mice were treated by oral gavage 5 days per week and metastases were monitored periodically by bioluminescence. Mice were euthanized at day 28 after the arterial injection of tumor cells.

### Autophagy assessment in vivo by Western blot analysis

Lung tissues harvested at necropsy were processed into protein lysates by homogenization (Bio-Gen PRO200, PRO Scientific Inc., Oxford, CT) and sonication (50 watt sonic dismembrator, Fisher Scientific, Hampton, NH) in T-PER® Tissue Protein Extraction Reagent (Thermo Fisher Scientific) with cOmplete™ EDTA-free protease inhibitor cocktail and PhosSTOP phosphatase inhibitors (Sigma-Aldrich) according to manufacturer’s instructions. Following centrifugation to remove insoluble material, protein levels in soluble lysates were quantified using the DC protein assay (Bio-Rad) and 15 µg/lane was loaded onto precast 4–20% Mini-Protean and Criterion TGX gels (Bio-Rad). Immunoblotting was performed as described for the cell lines.

### Statistical analysis

GraphPad Prism software was used for evaluating statistical differences between treatments. Student’s *t*-test was applied for pairwise comparisons. For assessing multiple comparisons (e.g., inhibition of viability data) we used ANOVA with Tukey’s post-hoc test. *p*-Values of < 0.05 were considered significant.

### Reporting summary

Further information on research design is available in the Nature Research Reporting Summary linked to this article.

## Supplementary information

Supplementary Information

Reporting Summary

## Data Availability

The data that support the findings of this study are available from the corresponding author upon reasonable request.
