# Supplementary material for: Targeting lonidamine to mitochondria mitigates lung tumorigenesis and brain metastasis
Source: Nat Commun. 2019 May 17;10:2205. doi: 10.1038/s41467-019-10042-1 (PMC6525201; doi:10.1038/s41467-019-10042-1)
Supplement: Supplementary file 1 — Supplementary Information [file 41467_2019_10042_MOESM1_ESM.pdf]

Supplementary Information

Targeting Lonidamine to Mitochondria Mitigates Lung Tumorigenesis and Brain Metastasis

Cheng et al.

Supplementary Methods

Synthesis Mito-LND. All chemicals and organic solvents were commercially available and were used as supplied. The reactions were monitored by TLC using silica gel Merck 60F254. Crude materials were purified by flash chromatography on Merck Silica gel 60 (0.040–0.063 mm). ^{31}P NMR, ^{1}H NMR and ^{13}C NMR spectra were recorded at 121.49, 400.13 and 75.54 MHz respectively using a Bruker DPX AVANCE 400 spectrometer equipped with a QNP probe. ^{31}P NMR was taken in $CDCl_3$ using 85% H_3PO_4 as an external standard with broad-band ^{1}H decoupling. ^{1}H NMR and ^{13}C NMR were taken in $CDCl_3$ using TMS or $CDCl_3$ as internal reference respectively. Chemical shifts (δ) are reported in ppm and coupling constant *J* values in Hertz.

Mito-LND was synthesized as follows (Supplementary Figure 14): A solution of 1-(2, 4-dichlorobenzyl)-1H-indazole-3-carboxylic acid (0.35 g, 1.1 mmol), oxalyl chloride (2 mL), and a catalytic amount of DMF (0.1 mL) in CH_2Cl_2 (20 ml) was heated under reflux for 2 h. Unreacted oxalyl chloride and solvent were removed under reduced pressure to yield 1-(2,4-dichlorobenzyl)-1H-indazole-3-carbonyl chloride as a yellow solid. (10-aminodecyl)-triphenylphosphonium bromide (0.54 g, 1.1 mmol) and trietylamine (160 µL, 1.1 mmol) were added to a solution of acyl chloride in CH_2Cl_2 (20 mL), and the reaction mixture was stirred for 12 h at room temperature and then washed with water (30 mL). The organic layer was dried over Na_2SO_4 and the solvent distilled under reduced pressure. Purification of the crude product by flash chromatography on a silica gel (CH_2Cl_2/EtOH 90:10) afforded a yellow powder (0.4 g, 46%), corresponding to Mito-LND.

The NMR data for Mito-LND are as follows:

^{31}P (121.49 MHz, $CDCl_3$) δ 24.54. ^{1}H NMR (400.13 MHz, $CDCl_3$) δ 8.40 (1H, d, *J* = 8.1), 7.88-7.78 (9H, m), 7.74-7.69 (6H, m), 7.43 (1H, d, *J* = 2.2), 7.42-7.33 (2H, m), 7.30-7.26 (1H, m), 7.1 (1H, dd, *J* = 2.0, 8.3), 7.03 (1H, bt, *J* = 5.9), 6.66 (1H, d, *J* = 8.3), 5.66 (2H, s), 3.78-3.71 (2H, m), 3.45 (2H, dt, *J* = 6.8, 7.1), 1.70-1.56 (6H, m), 1.31-1.17 (10H, m). ^{13}C NMR (75.47 MHz, $CDCl_3$) δ 162.4, 141.1, 138.6, 135.0, 134.9, 134.4, 133.7, 133.6, 133.1, 132.4, 130.5, 130.4, 129.4, 129.4, 127.6, 127.3, 123.1, 123.0, 122.8, 118.8, 117.9, 109.2, 50.0, 39.0, 30.4, 30.3, 29.7, 29.3, 29.2, 29.1, 29.0, 26.9, 23.0, 22.7, 22.6, 22.5.

The high resolution mass spectrometry (HRMS) data calculated for Mito-LND $C_{43}H_{45}Cl_2N_3OP$ $[M]^+$: 720.2672, found: 720.2672

Synthesis Alkyl-lonidamine (Alkyl-LND).

A solution of 1-(2,4-dichlorobenzyl)-1H-indazole-3-carboxylic acid (0.35 g, 1.1 mmol), oxalyl chloride (2 mL) and a catalytic amount of DMF (0.1 mL) in CH_2Cl_2 (20 mL) was heated under reflux for 2 h. Unreacted oxalyl chloride and solvent were removed under reduced pressure to yield 1-(2,4-dichlorobenzyl)-1H-indazole-3-carbonyl chloride as a yellow solid. 10-Aminodecane (0.17 g, 1.1 mmol) and triethylamine (160 mL, 1.1 mmol) were added to a solution of the acid chloride in CH_2Cl_2 (10 mL) and the reaction mixture was stirred for 12 h at room temperature and then washed with water (30 mL). The organic layer was dried over Na_2SO_4 and the solvent distilled under reduced pressure. Purification of the crude product by flash chromatography on a silica gel (using CH_2Cl_2) afforded a white powder (0.4 g, 80%), corresponding to **Alkyl-lonidamine (Alkyl-LND)**.

The NMR data for Alkyl-LND are as follows:

^{1}H NMR (400.13 MHz, $CDCl_3$) d 8.44 (1H, d, *J* = 8.2), 7.45 (1H, d, 1.8),7.43-7.29 (3H, m), 7.11 (1H, dd, *J* = 8.3; 1.8), 7.01 (1H, t, *J* = 5.0), 6.62 (1H, dd, *J* = 8.4), 5.67 (2H, s), 3.49 (2H, dt, *J* = 6.9, 13.7), 1.70-1.61 (4H, m), 1.44-1.27 (12H, m), 0.88 (3H, t, *J* = 6.5). ^{13}C NMR (75.47 MHz, $CDCl_3$) d 162.3, 141.2, 138.7, 134.4, 133.1, 132.5, 129.4, 129.2, 127.6, 127.4, 123.1, 123.0, 122.9, 109.1, 50.0, 39.1, 31.9, 29.8, 29.5, 29.3, 29.2, 27.0, 22.6, 14.1.

The HRMS data calculated for **Alkyl-LND** $C_{25}H_{31}Cl_2N_3O$ $[M+H]^+$: 460.1917, found: 460.1917.

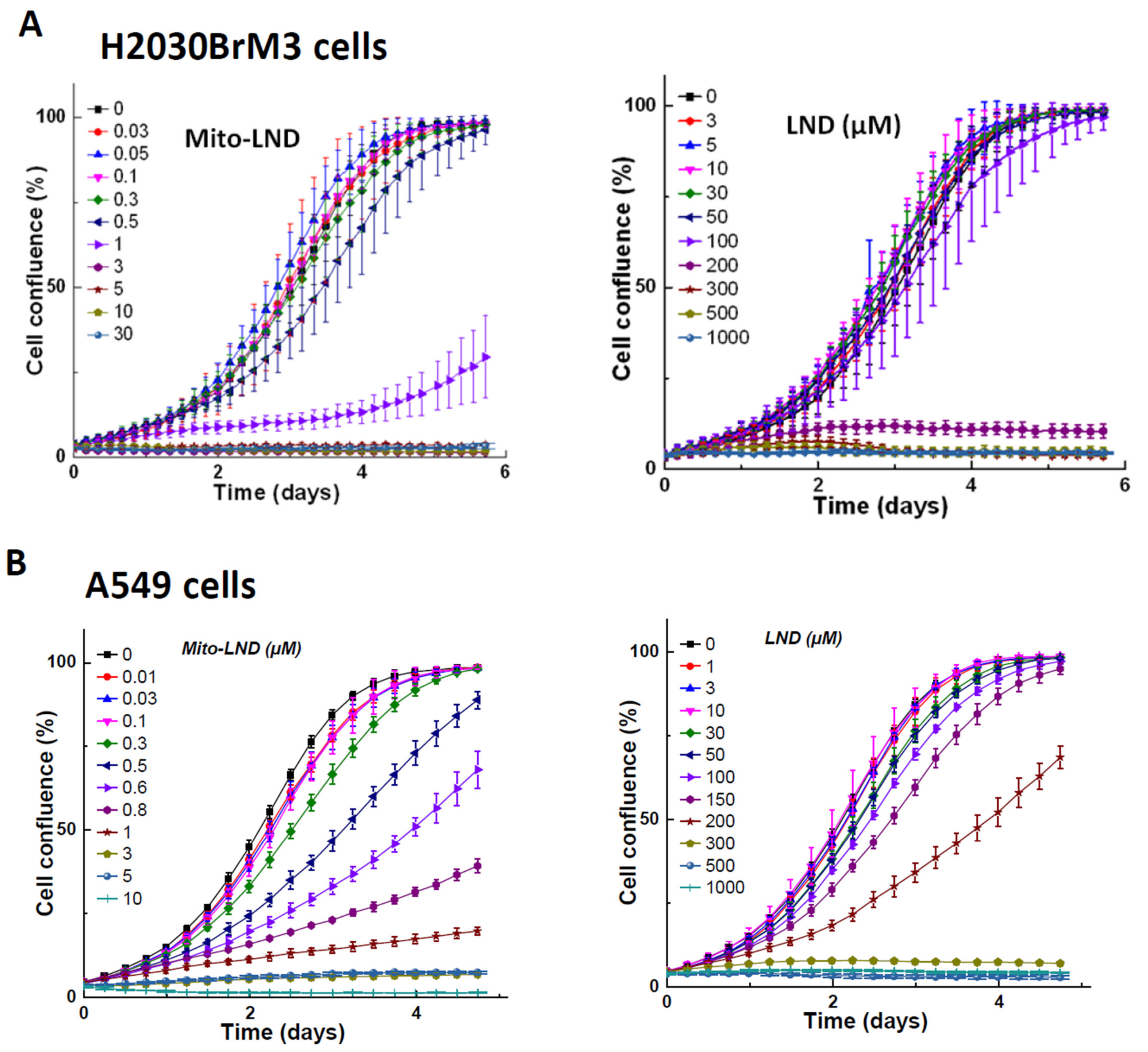

Supplementary Figure 1. Effect of Mito-LND on lung cancer H2030BrM3 and A549 cell proliferation. H2030BrM3 **A.** and A549 **B.** cells were treated with LND or Mito-LND. Cell proliferation was monitored in real-time with the continuous presence of indicated treatments until the end of each experiment. Dose response effects of LND and Mito-LND on cell confluence are shown. The cell confluence data were used to determine the IC_{50} values, as shown in Figure 1B.

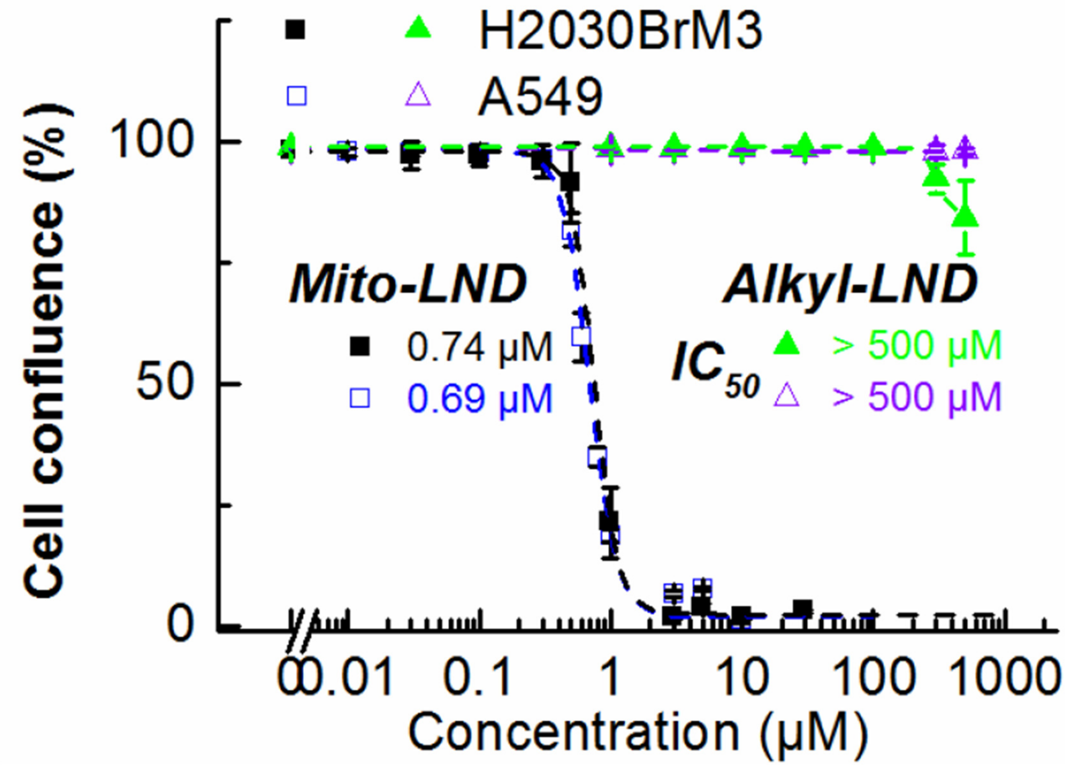

Supplementary Figure 2. Comparison of the effects of Alkyl-LND and Mito-LND on the proliferation of lung cancer cells. H2030BrM3 or A549 cells were treated with Alkyl-LND or Mito-LND. Cell proliferation was monitored in real-time with the continuous presence of the indicated treatments until the end of each experiment. The cell confluence (as control groups reach 98% confluency) is plotted against concentration. Dashed lines represent the fitting curves used to determine the IC_{50} values, as indicated.

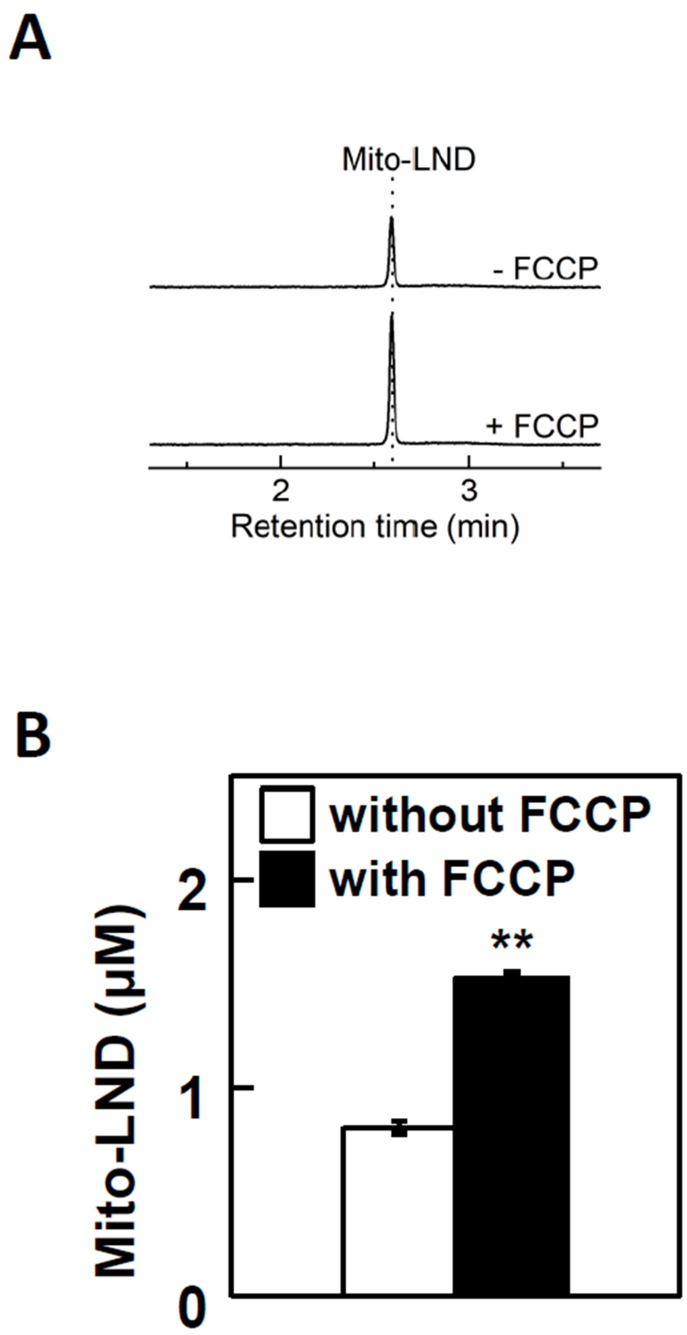

Supplementary Figure 3. Uptake of Mito-LND into isolated mitochondria. A suspension of isolated mitochondria (1.5 mg/mL) was incubated with Mito-LND (10 μM) for 2 min, followed by an additional 2 min incubation in the presence or absence of the mitochondrial uncoupler, FCCP (1 μM). Mito-LND concentration in the supernatant was measured by HPLC of the medium after mitochondria had been pelleted, as described in the Materials and Methods section. **A.** HPLC fluorescence traces recorded; **B.** Quantitative analyses of Mito-LND concentrations. Data are presented as the means ± SD, t test versus control: $^{**}p<0.01$

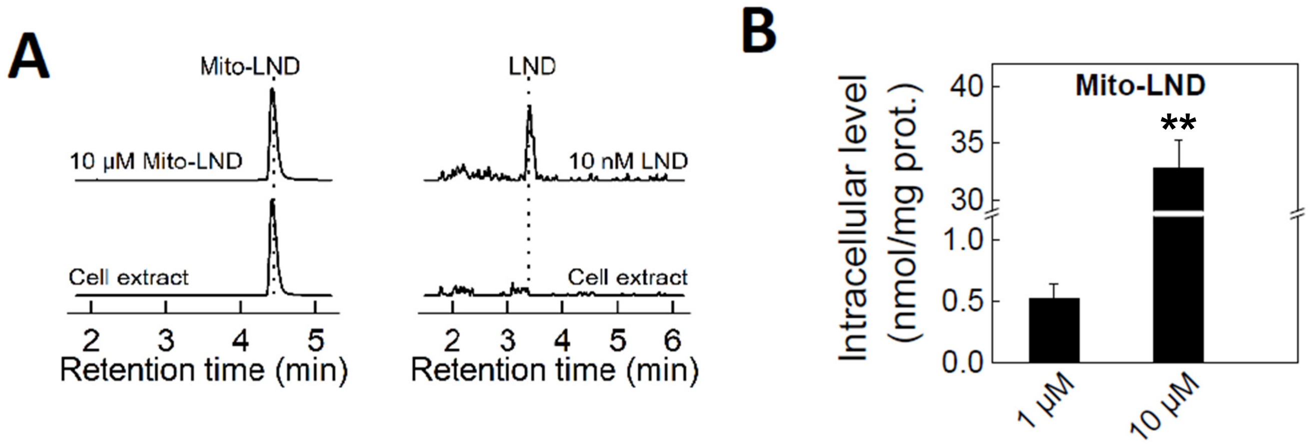

Supplementary Figure 4. Mito-LND is not metabolized to release LND in lung cancer cells. H2030BrM3 cells were treated with Mito-LND (1 or 10 µM) for 24 h and the intracellular levels of Mito-LND and LND were determined by LC-MS/MS analyses of cell extracts, as described in the Materials and Methods section. **A.** Representative LC-MS/MS traces of Mito-LND (MRM transition: 720.20>262.00) and LND (MRM transition: 319.10>274.95). **B.** Quantitative data on intracellular levels of Mito-LND. Data are presented as the means ± SD, t test versus control: **p<0.01

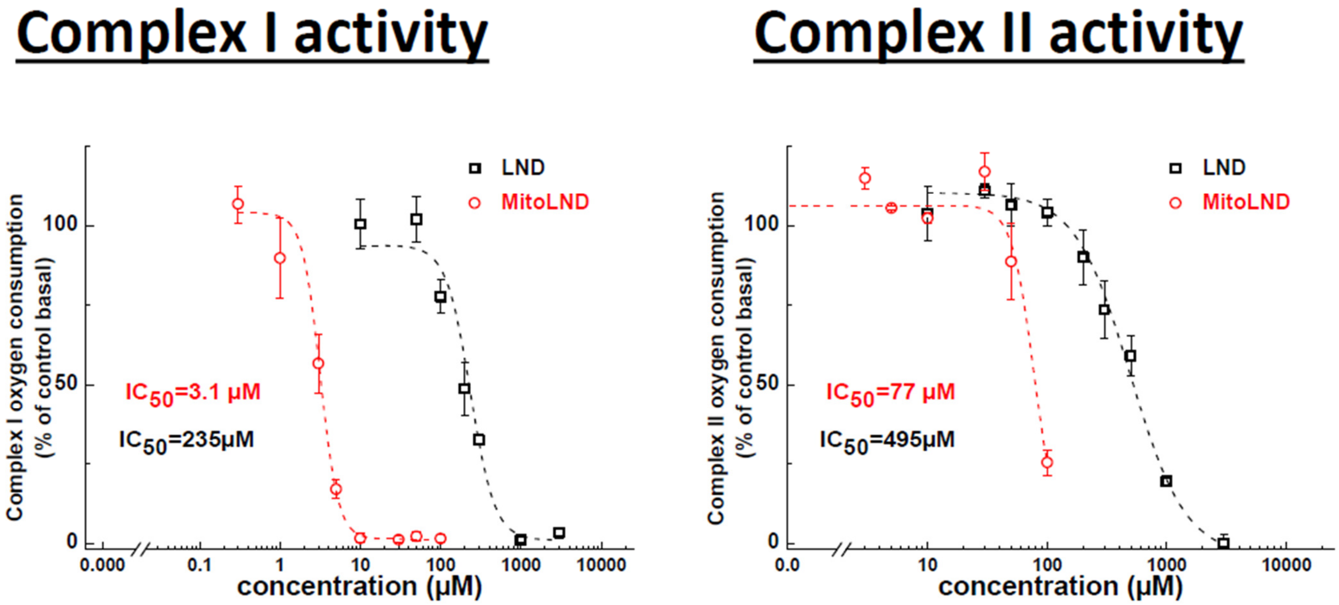

Supplementary Figure 5. Effect of LND and Mito-LND on the oxygen consumption rate measured immediately after their addition to H2030BrM3 cells. LND and Mito-LND directly inhibit both pyruvate-driven and succinate-driven respiration in permeabilized cells. H2030BrM3 cells were permeabilized in mannitol and sucrose (MAS) buffer containing assay media for either complex I (10 mM pyruvate and 1.5 mM malate as substrates of complex I and 10 mM malonate as complex II inhibitor) or complex II activities (10 mM succinate as a substrate of complex II and 1 μM rotenone as complex I inhibitor). Either LND or Mito-LND was added acutely to permeabilized cells and oxygen consumption rates (OCR) were assayed immediately. Complex I and complex II-mediated oxygen consumption rates were plotted against the concentration of LND or Mito-LND, and IC_{50} values determined. Dashed lines represent the fitting curves used for determination of the IC_{50} values.

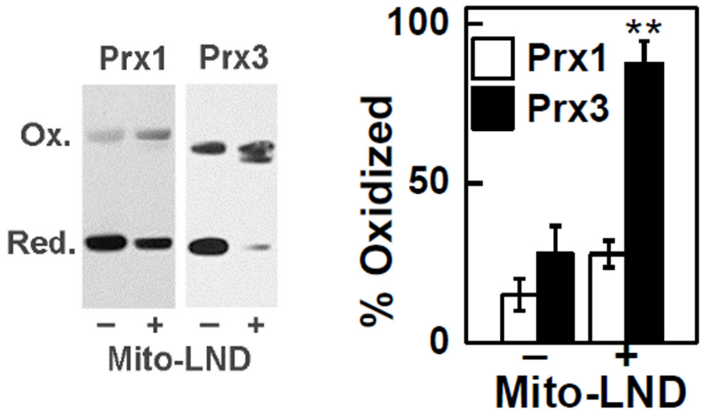

Supplementary Figure 6. Effect of LND and Mito-LND on redox status of cytosolic (Prx1) and mitochondrial (Prx3) peroxiredoxins in H2030BrM3 cells. Representative immunoblots from cells treated with Mito-LND (0.3 µM, 24 h) are shown in the left panel, while the quantitative analyses of the redox status of Prx1 and Prx3 are shown in the right panels. Data are presented as the means ± SD, t test versus control: **p<0.01.

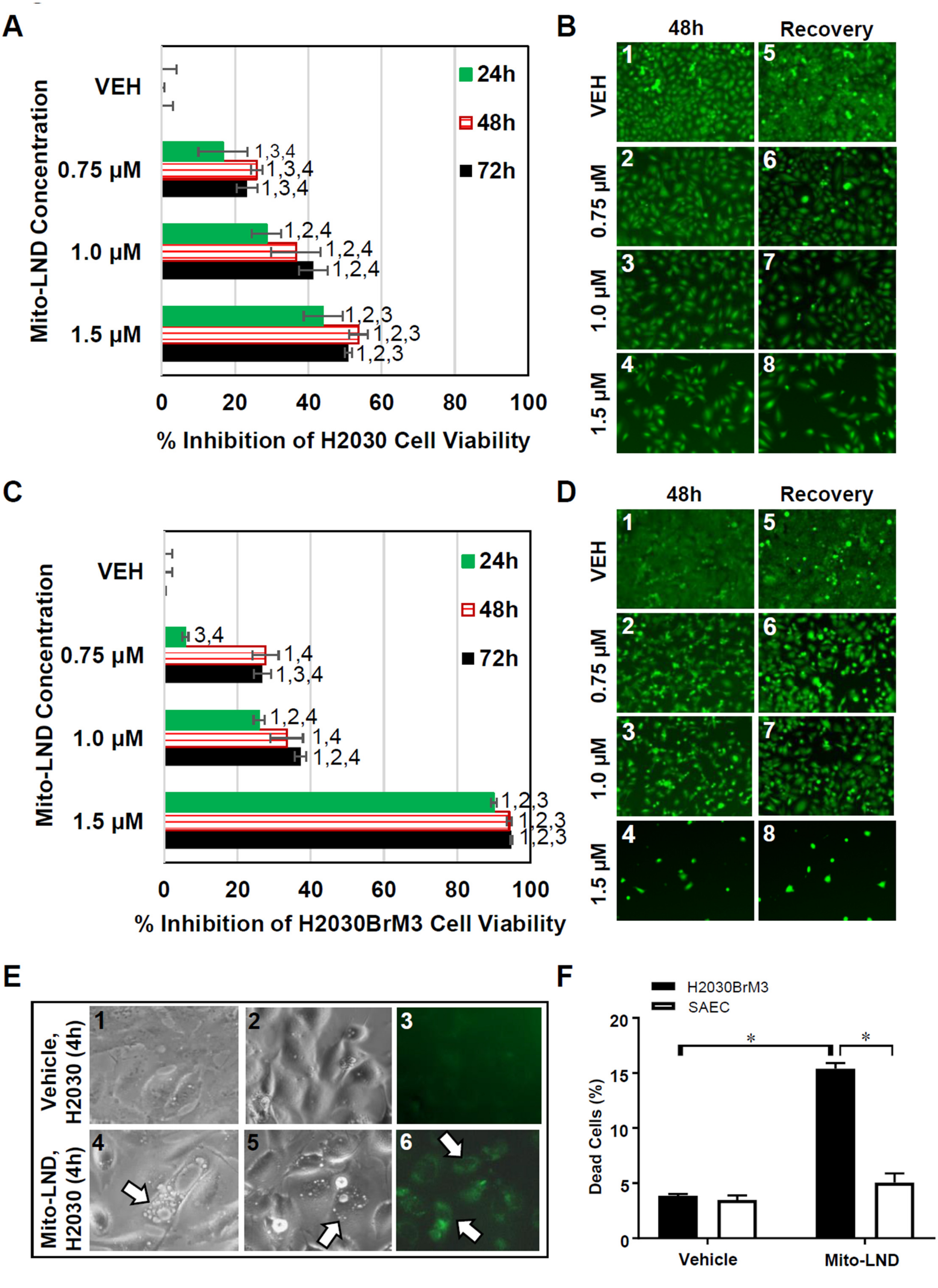

A
Mito-LND Concentration
VEH
0.75 µM
1.0 µM
1.5 µM
24h
48h
72h
1,3,4
1,2,4
1,2,3
0 20 40 60 80 100
% Inhibition of H2030 Cell Viability
B
48h
Recovery
1 2 3 4 5 6 7 8
C
Mito-LND Concentration
3,4
1,4
1,3,4
1,2,4
1,2,3
% Inhibition of H2030BrM3 Cell Viability
D
E
Vehicle, H2030 (4h)
Mito-LND, H2030 (4h)
F
H2030BrM3
SAEC
Dead Cells (%)
0 5 10 15 20
*
Vehicle
Mito-LND

Supplementary Figure 7. Mito-LND induces autophagic cell death resulting in reduced cell viability. A. Mito-LND (2 µM) inhibits H2030 cell viability ($P<0.05$, based on two-way ANOVA with Tukey's post-hoc test for multiple comparisons), [1]significantly different from Vehicle, [2]significantly different from Mito-LND (0.75 µM), [3]significantly different from Mito-LND (1.0 µM), and [4]significantly different from Mito-LND (1.5 µM). Data are presented as the mean ± SEM. **B.** Representative H2030 cell images 48 h post vehicle or Mito-LND treatment (1-4) or 48 h post-recovery of treated cells (48 h of treatment, followed by fresh media replenishment and growth for an additional 48 h (5-8); 1) Vehicle, 2) Mito-LND (0.75 µM), 3) Mito-LND (1.0 µM), 4) Mito-LND (1.5 µM). **C.** Mito-LND (2 µM) inhibits H2030BrM3 cell viability ($P<0.05$, based on two-way ANOVA with Tukey's post-hoc test for multiple comparisons), [1]significantly different from Vehicle, [2] significantly different from Mito-LND (0.75 µM), [3]significantly different from Mito-LND (1.0 µM), and [4]significantly different from Mito-LND (1.5 µM). Data are presented as the mean ± SEM. **D.** Representative H2030BrM3 cell images 48 h post vehicle or Mito-LND treatment (1-4) or 48 h post-recovery of treated cells (48 h of treatment, followed by fresh media replenishment and growth for an additional 48 h (5-8); 1) Vehicle, 2) Mito-LND (0.75 µM), 3) Mito-LND (1.0 µM), 4) Mito-LND (1.5 µM). **E.** Mito-LND induces autophagy in H2030 cells treated with Vehicle (in panels 1-3) or Mito-LND (2 µM) (in panels 4-6); MDC staining for detection of acidic autophagic vacuoles shown in "3" for Vehicle and "6" for Mito-LND treated H2030 cells, arrows indicate autophagic vacuoles in bright field images of Mito-LND treated cells (E2 and E5) and matched FL monodansylcadaverine labeled vacuoles (E3 and E6). **F.** Effects of Mito-LND on cell death in normal lung cells (SAEC) and lung cancer cells (H2030BrM3). Cells were treated with Mito-LND (2 µM) for 24 h and cell death was measured by flow cytometric analysis. Data are presented as the means ± SEM, n=5 or 6, t test versus control: *$p<0.05$.

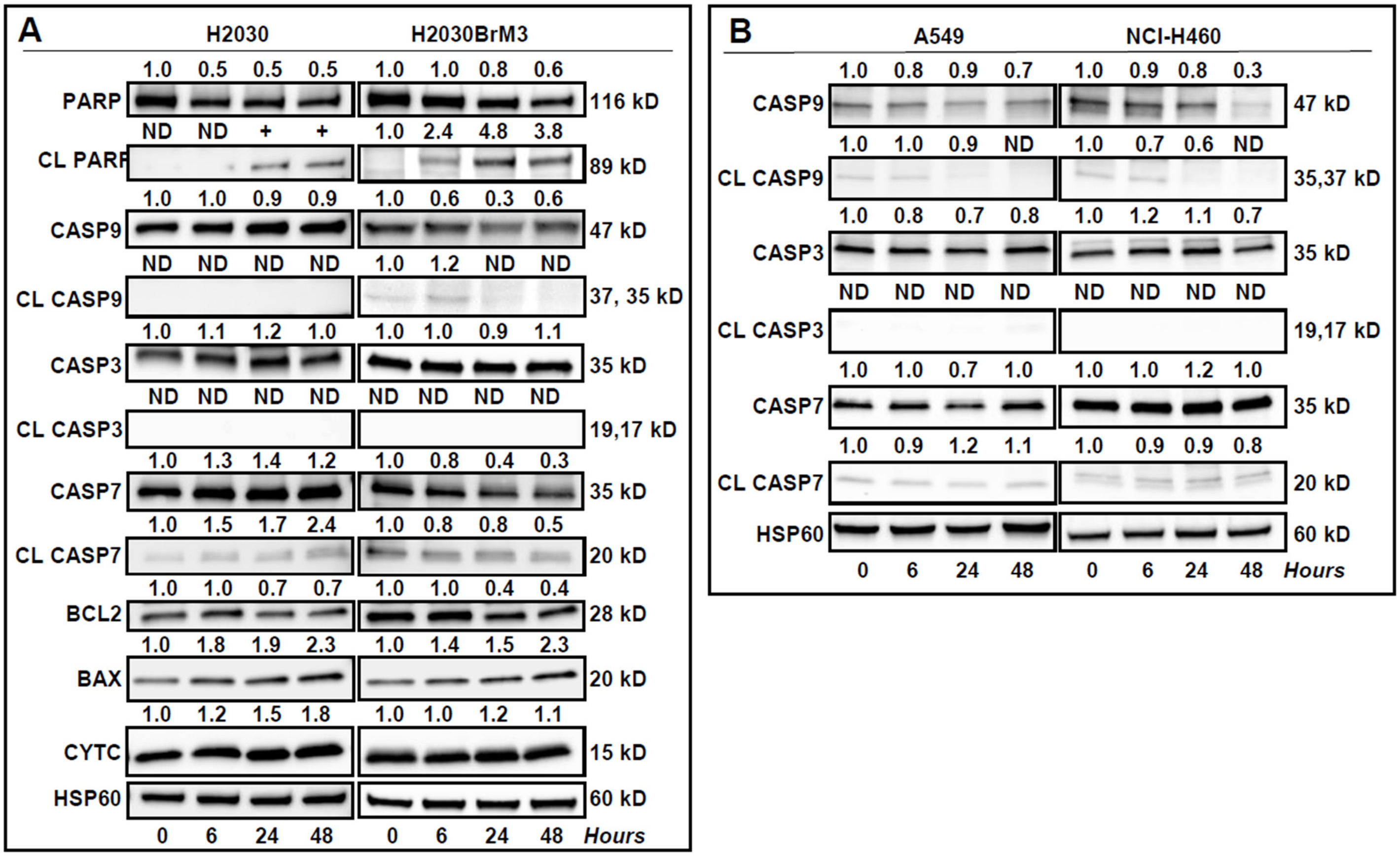

Supplementary Figure 8. The effect of Mito-LND on markers of apoptosis in H2030 and H2030BrM3 lung cancer cell lines over time. A. Results from Mito-LND-treated H2030 and H2030BrM3 cells indicate that cell death induction is mainly via autophagy rather than caspase-dependent apoptosis. Mito-LND treatment (2 µM) resulted in increased levels of cleaved PARP and cytochrome *c*, but did not induce caspase cleavage. Additionally, Mito-LND increased levels of pro-death BAX and reduced BCL-2 levels consistent with autophagic cell death. **B.** Analogous data obtained for A549 (*left*) and NCI-H460 (*right*) lung cancer cells.

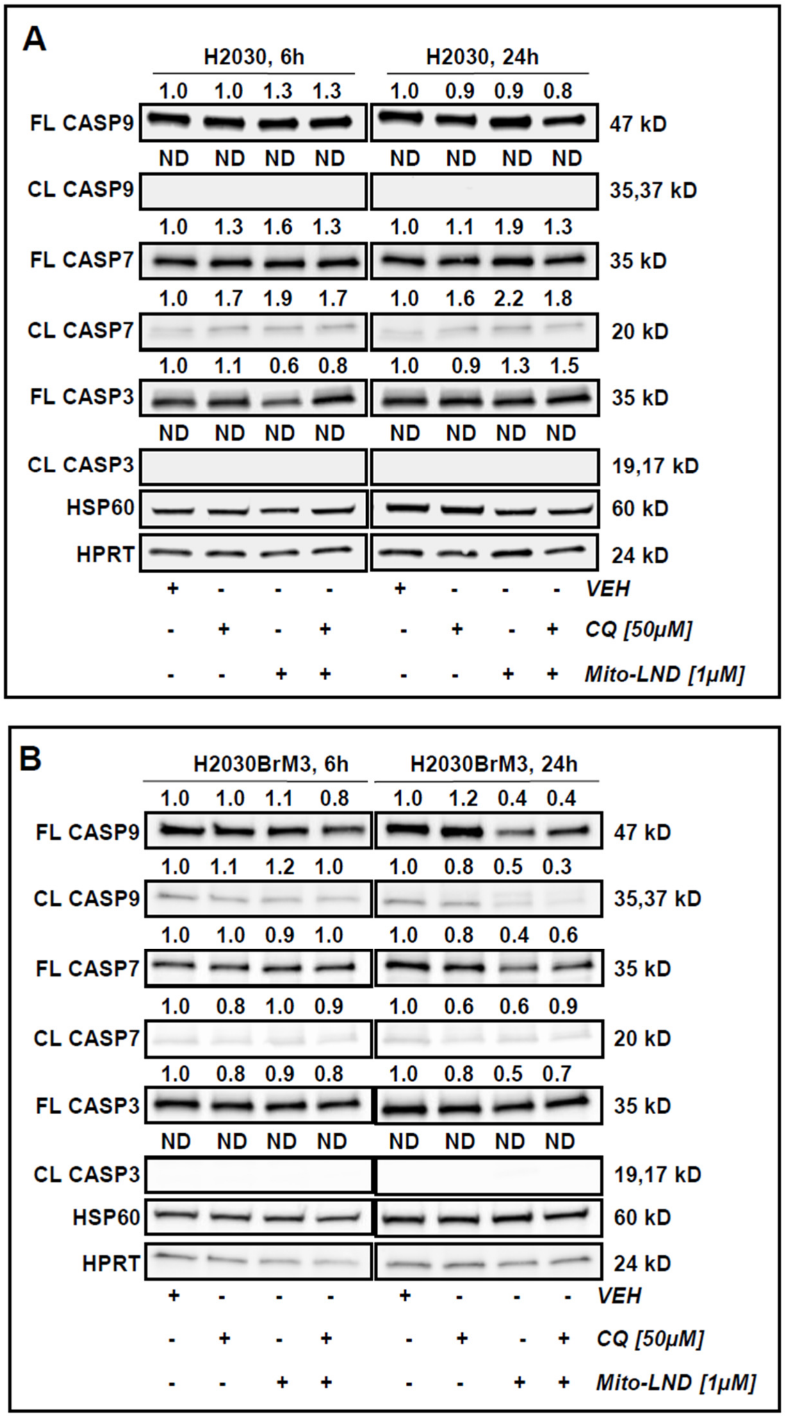

Supplementary Figure 9. Effects of inhibition of autophagy on Mito-LND-induced caspase alterations in H2030 and H2030BrM3 lung cancer cell lines. A. Treatment of H2030 cells with Mito-LND or chloroquine (CQ) alone or in combination does not induce cleavage of CASP9 or CASP3, but mildly induces cleavage of CASP7. **B.** Treatment of H2030BrM3 cells with Mito-LND or chloroquine alone or in combination does not induce CASP cleavage, but mildly reduces cleavage of CASP7 and CASP9 at 24 h.

A

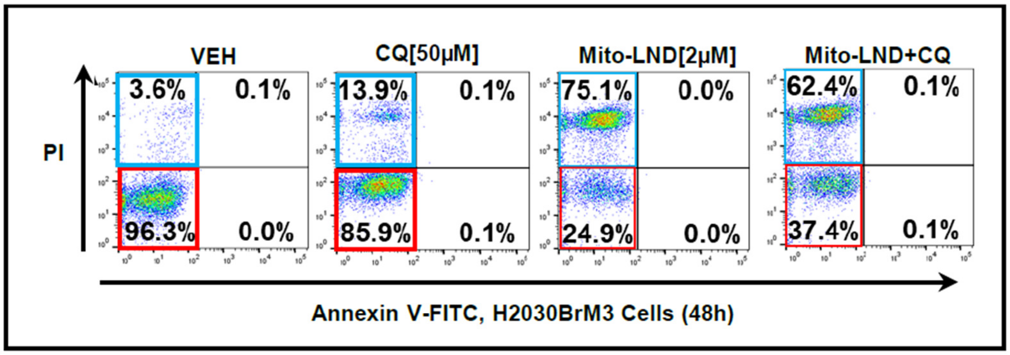

B

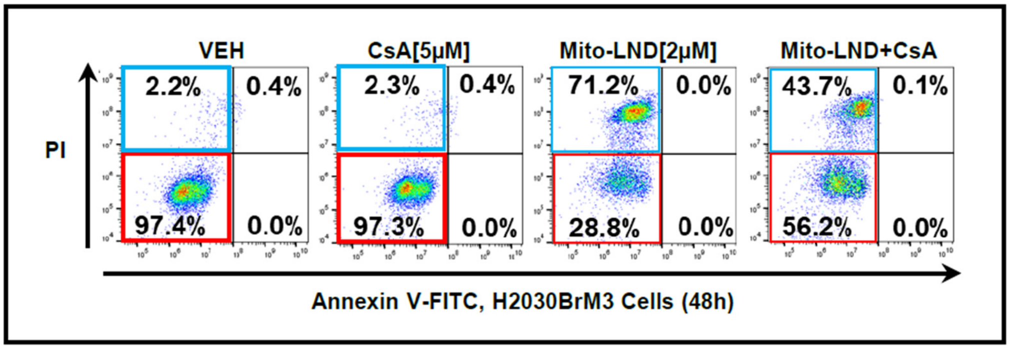

C

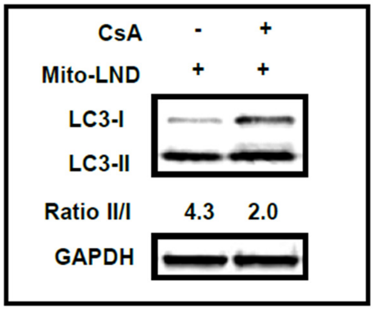

D

Treatment (48h)	% Live	% Early Apoptotic	% Late Apoptotic	% Dead & Necrotic
Vehicle[a]	96.3	0.0	0.1	3.6
2μM Mito-LND[b]	24.9[a,c,d]	0.0	0.0	75.1[a,c,d]
50μM Chloroquine[c]	85.9[a,b,d]	0.1	0.1	13.9[a,b,d]
50μM Chloroquine + 2μM Mito-LND[d]	37.5[a,b,c]	0.1	0.1	62.4[a,b,c]

E

Treatment (48h)	% Live	% Early Apoptotic	% Late Apoptotic	% Dead & Necrotic
Vehicle[a]	97.4	0.0	0.4	2.2
2μM Mito-LND[b]	28.8[a,c,d]	0.0	0.0	71.2[a,c,d]
5μM Cyclosporin A[c]	97.3[b,d]	0.0	0.4	2.3[b,d]
5μM Cyclosporin A + 2μM Mito-LND[d]	56.2[a,b,c]	0.0	0.1	43.7[a,b,c]

Supplementary Figure 10. Mito-LND induces non-apoptotic cell death in lung cancer cell lines which is blocked by autophagy inhibitors. A. Pretreatment of H2030BrM3 cells with CQ, an inhibitor of late autophagy, significantly reduces Mito-LND induced cell death (*upper left* (*UL*) quadrant, blue

outline), increases live cells (*lower left* (*LL*) quadrant, red outline) and has no impact on early apoptosis (*lower right* (*LR*) quadrant) or late apoptosis (*upper left* (*UL*) quadrant). **B.** Pretreatment of H2030BrM3 cells with CsA, a specific mitophagy inhibitor, significantly reduces Mito-LND induced cell death (*UL*), increases live cells (*LL*) and does not induce early (*LR*) or late apoptosis (*UL*). **C.** Mito-LND treatment of H2030BrM3 cells (48 h) results in a 4.3-fold increase in the LC3-**II**/LC-**I** ratio indicating formation of the lipidated autophagic form. CsA pretreatment reduces the conversion of LC-**I** to LC3-**II** induced by Mito-LND. **D.** & **E.** Percentage of live, early apoptotic, late apoptotic and dead or dying cells by treatment ($P<0.05$, based on two-way ANOVA with Tukey's post-hoc test for multiple comparisons), [a]significantly different from Vehicle, [b]significantly different from Mito-LND (2.0 μM), [c]significantly different from chloroquine (50.0 μM) or cyclosporin A (5.0 μM), and [d]significantly different from Mito-LND (2 μM) and chloroquine (50.0 μM) or cyclosporin A (5.0 μM); similar results were noted 24 h post-treatment).

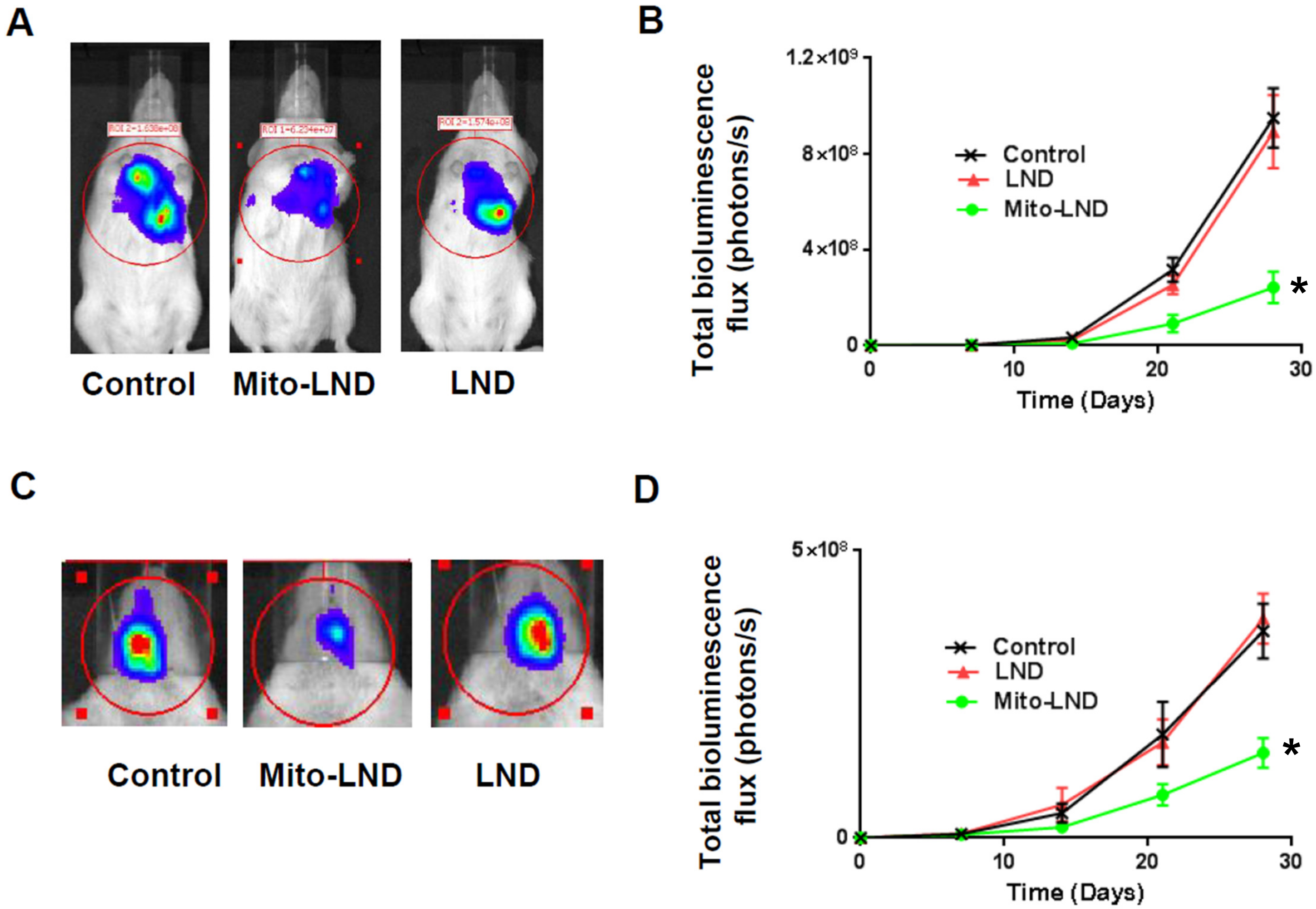

Supplementary Figure 11. Effect of Mito-LND on orthotopic lung tumor growth and brain metastases in mice injected with A549 cells. A & B. Data obtained from the orthotopic lung tumor model. **A.** Representative bioluminescence images from mice treated with either gavage control, LND or Mito-LND. **B.** Quantification of bioluminescence imaging signal intensity in the control, LND or Mito-LND treated groups at different time points after injection of A549 cells. **C & D**. Data obtained from the brain metastasis model. **C.** Representative bioluminescence images from mice treated with either gavage control, LND or Mito-LND. **D.** Quantification of bioluminescence imaging signal intensity in the control, LND or Mito-LND treated groups at different time points after injection of A549 cells. Quantified values are shown in total flux. Data are presented as the means ± SEM. Mito-LND treatment is statistically different than control or LND; test versus control or LND: *p<0.01.

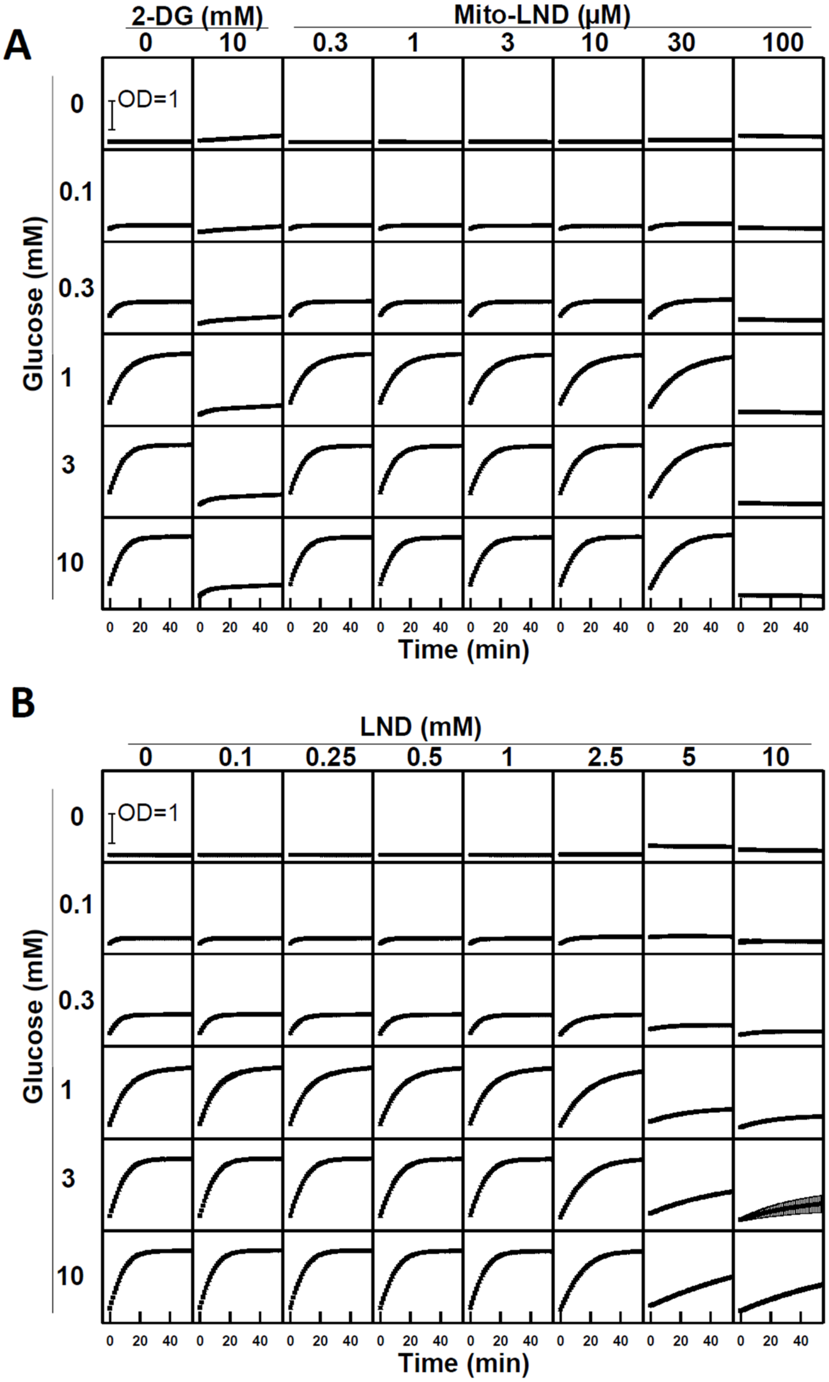

Supplementary Figure 12. The effect of Mito-LND and LND on hexokinase activity in enzyme-based assay measured in a 96-well plate format. The effects of 2-DG (positive control) and Mito-LND (panel A) and LND (panel B) on hexokinase activity. Hexokinase activity was measured with the glucose detection kit, as described in our previous publication (Cheng et al. Br. J. Cancer. 2014, 111(1):85-93). The assay vehicle was DMEM without glucose and FBS. O.D. corresponds to optical density (absorbance) at 340 nm due to NADH produced. Mito-LND and LND show hexokinase II inhibitory effects only at concentrations significantly (>50-fold) higher than those required to inhibit viability of lung cancer cells.

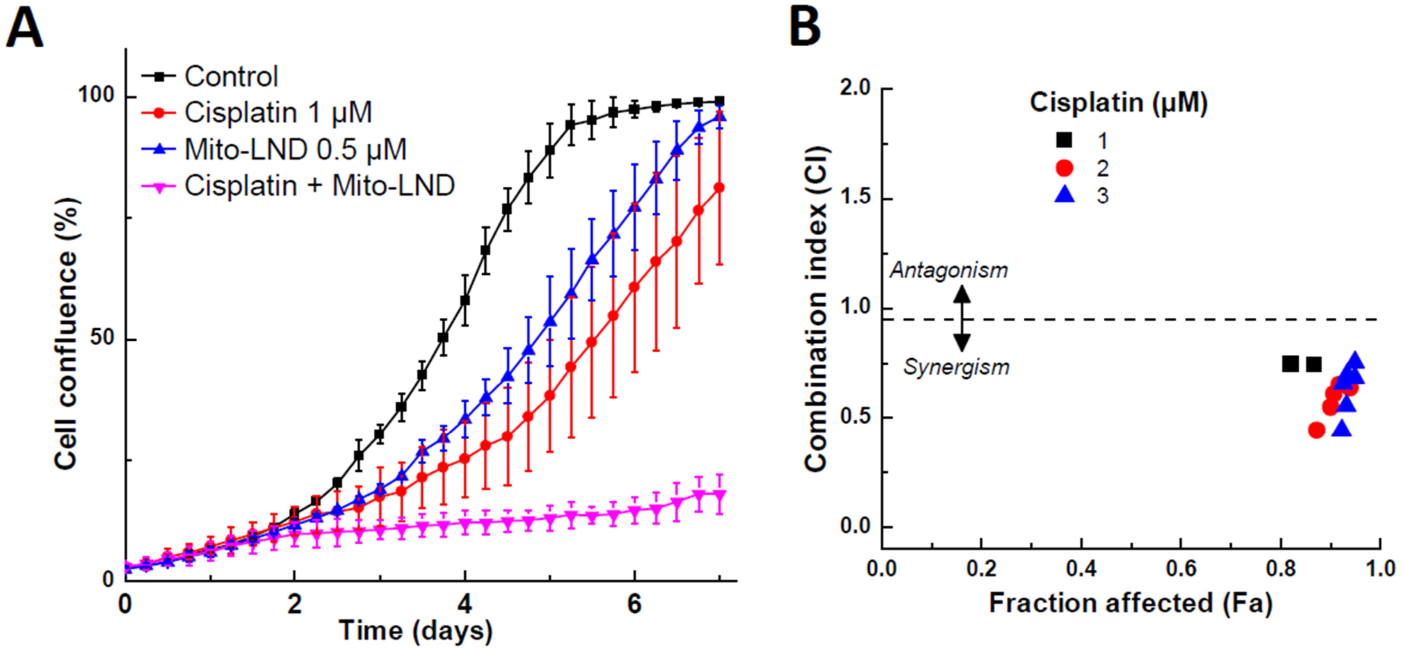

Supplementary Figure 13. Mito-LND synergistically sensitizes H2030BrM3 cancer cells to cisplatin. A. H2030BrM3 cells were treated with cisplatin (1 µM) or Mito-LND (0.5 µM) independently and together and cell growth was monitored continuously. Data shown are the mean ± SD (n=3). **B.** The combination index (CI)-fraction affected (Fa) plot for cells treated with 0.5 µM Mito-LND plus the three indicated concentrations of cisplatin. The fraction affected parameter is used as a measure of the drug(s) efficiency, with a value of 1 indicating complete inhibition of cell confluence and the value of zero indicating the lack of effect on cell confluence. CI values below 1 indicate synergistic interactions.

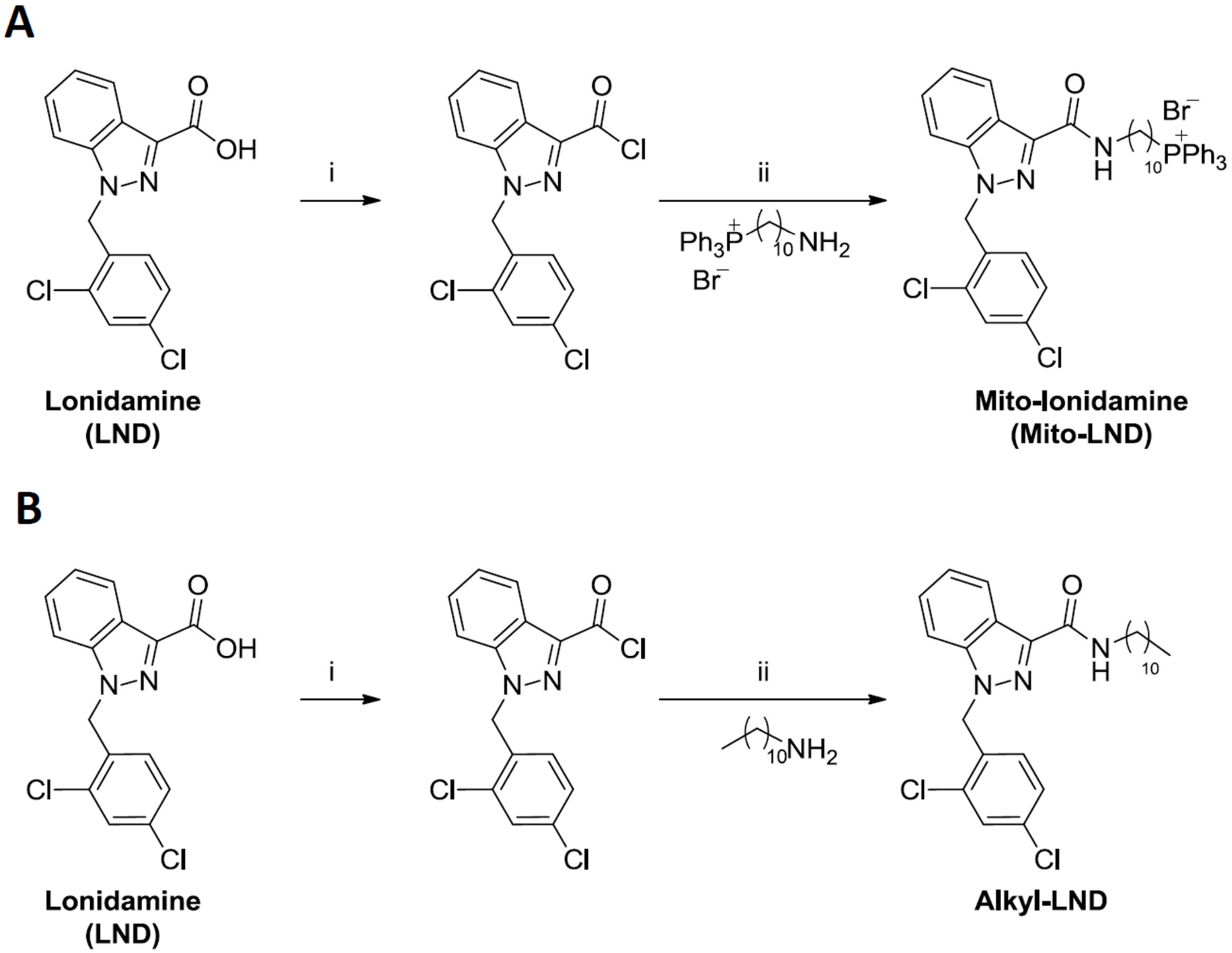

Supplementary Figure 14. A. Synthesis of Mito-LND. Reagents and conditions: i, $(COCl)_2$, CH_2Cl_2, DMF, reflux, 2 h; ii, TEA, CH_2Cl_2, 46%. **B. Synthesis of Alkyl-LND.** Reagents and conditions: i, $(COCl)_2$, CH_2Cl_2, DMF, reflux, 2 h; ii, TEA, CH_2Cl_2 80%.

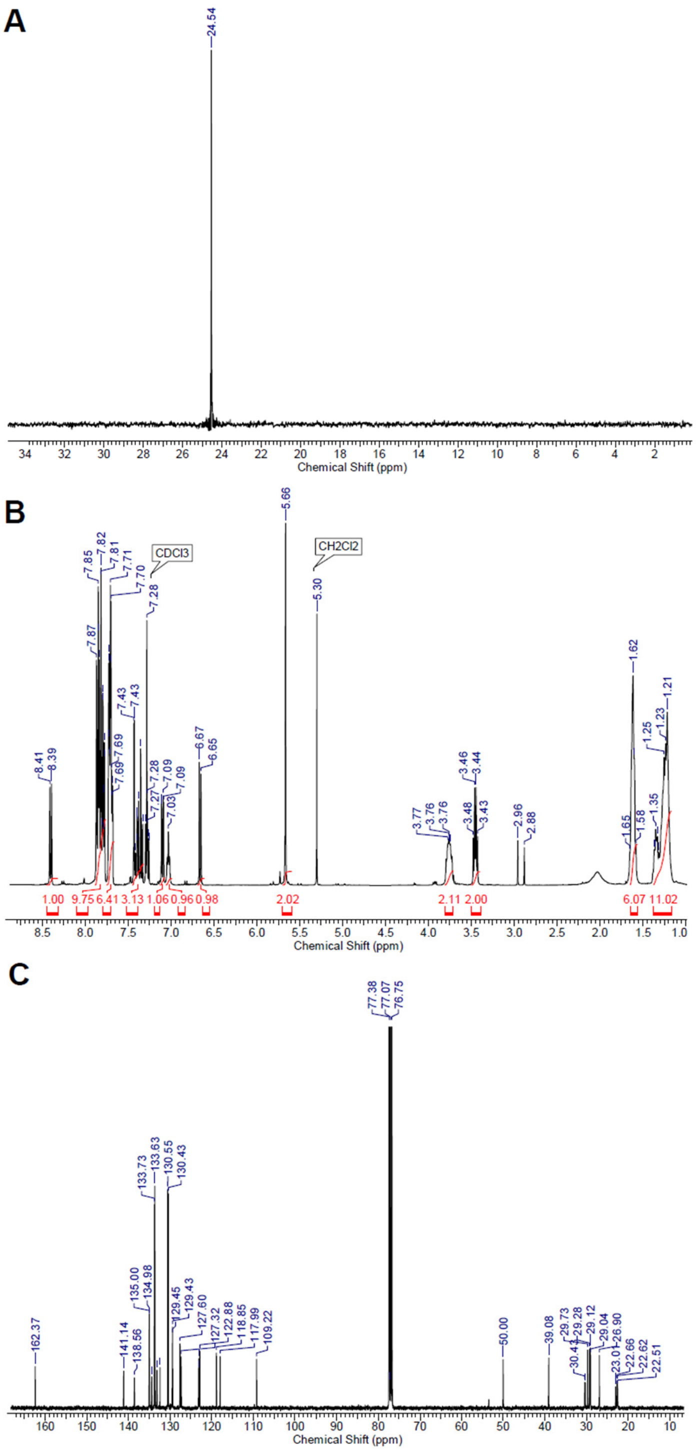

Supplementary Figure 15. A. Phosphorus (^{31}P) NMR spectrum of Mito-LND; B. proton (^{1}H) NMR spectrum of Mito-LND; C. Carbon (^{13}C) NMR spectrum of Mito-LND.

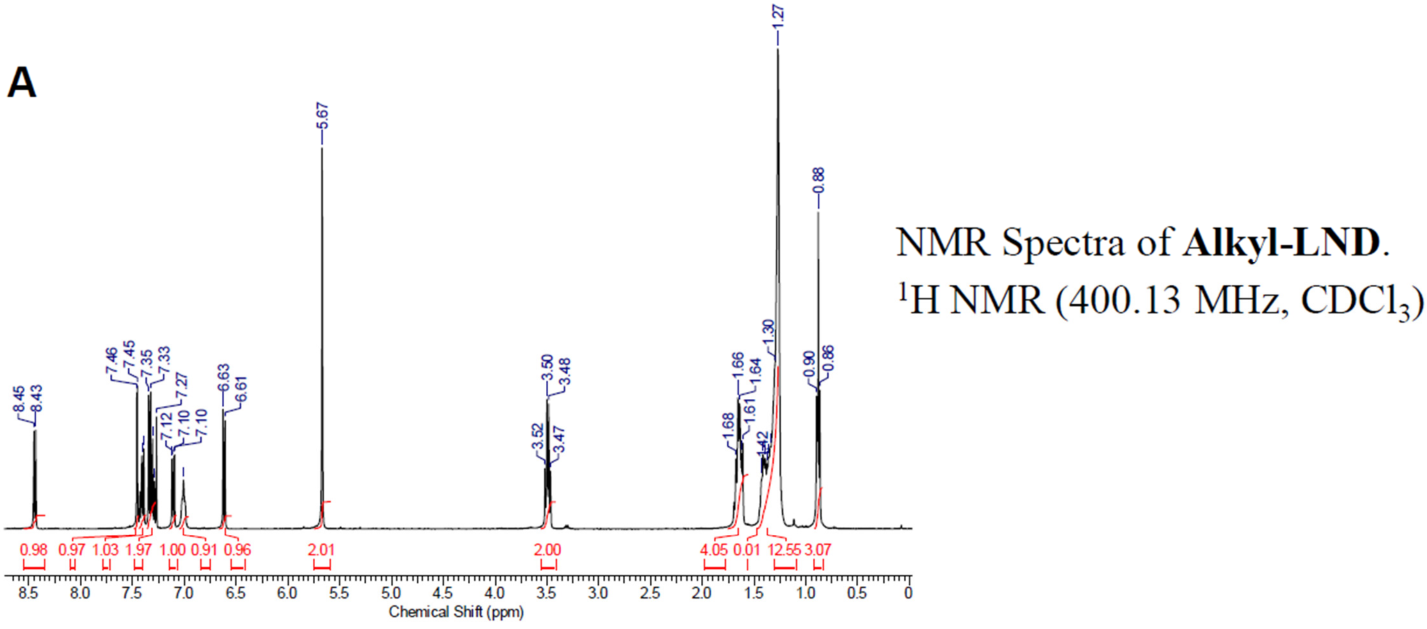

NMR Spectra of **Alkyl-LND**.

^{1}H NMR (400.13 MHz, $CDCl_3$)

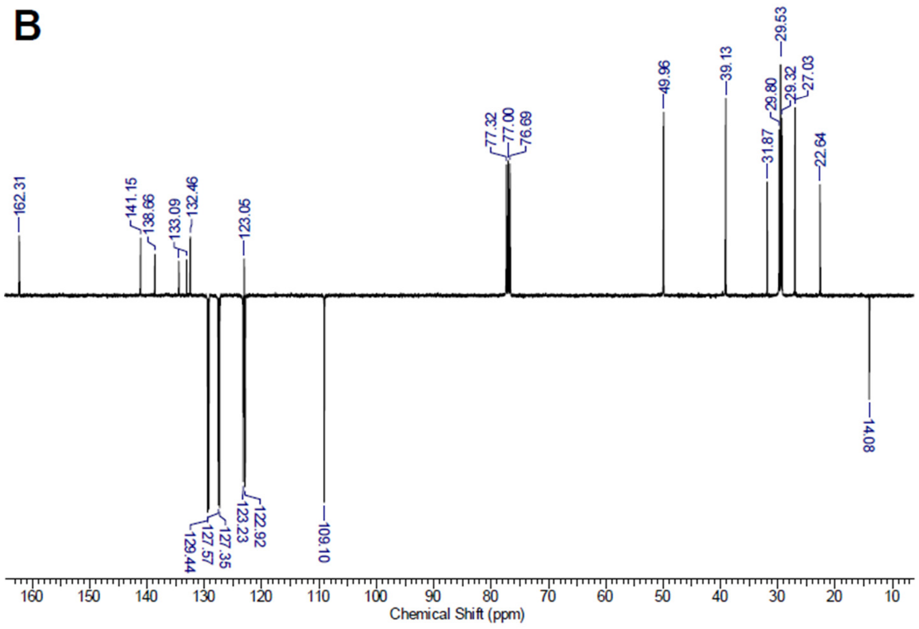

^{13}C (75 MHz, $CDCl_3$)

Supplementary Figure 16. A. Proton (^{1}H) NMR spectrum of Alkyl-LND; B. Carbon (^{13}C) NMR spectrum of Alkyl-LND.

Supplementary Figure 17. Full blots related to Figure 3A.

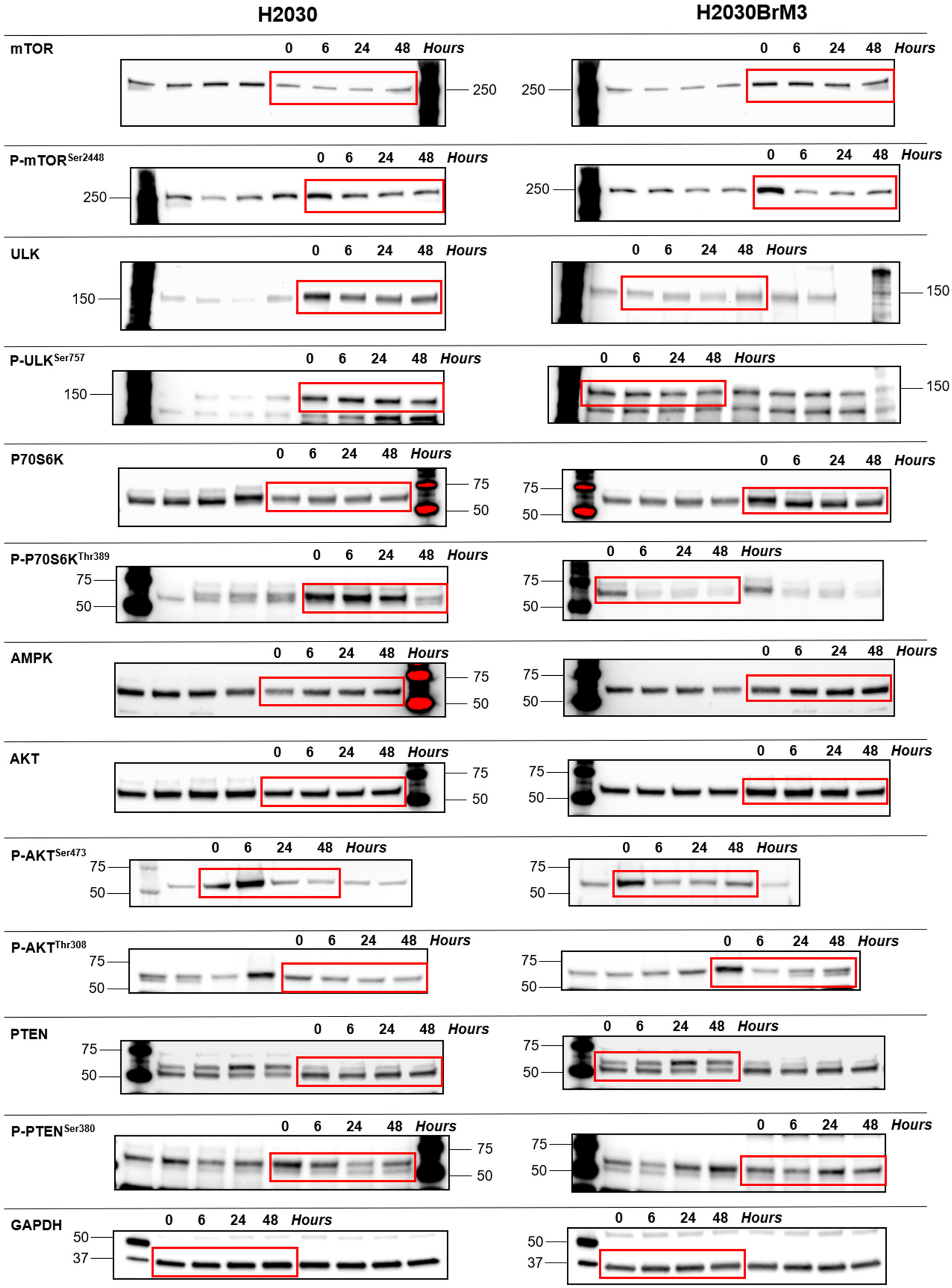

Supplementary Figure 18. Full blots related to Figure 3B.

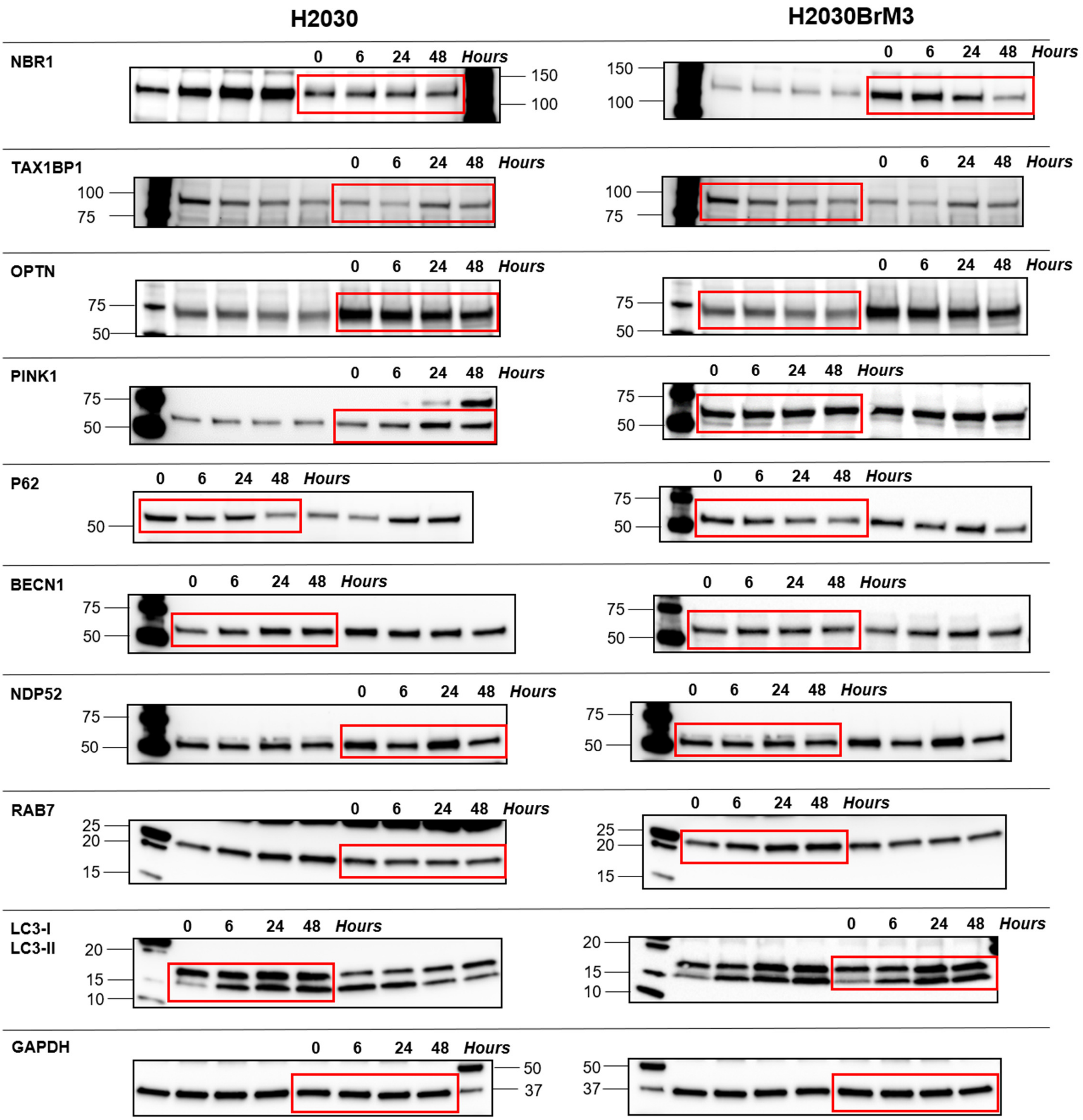

Supplementary Figure 19. Full blots related to Figure 3C.

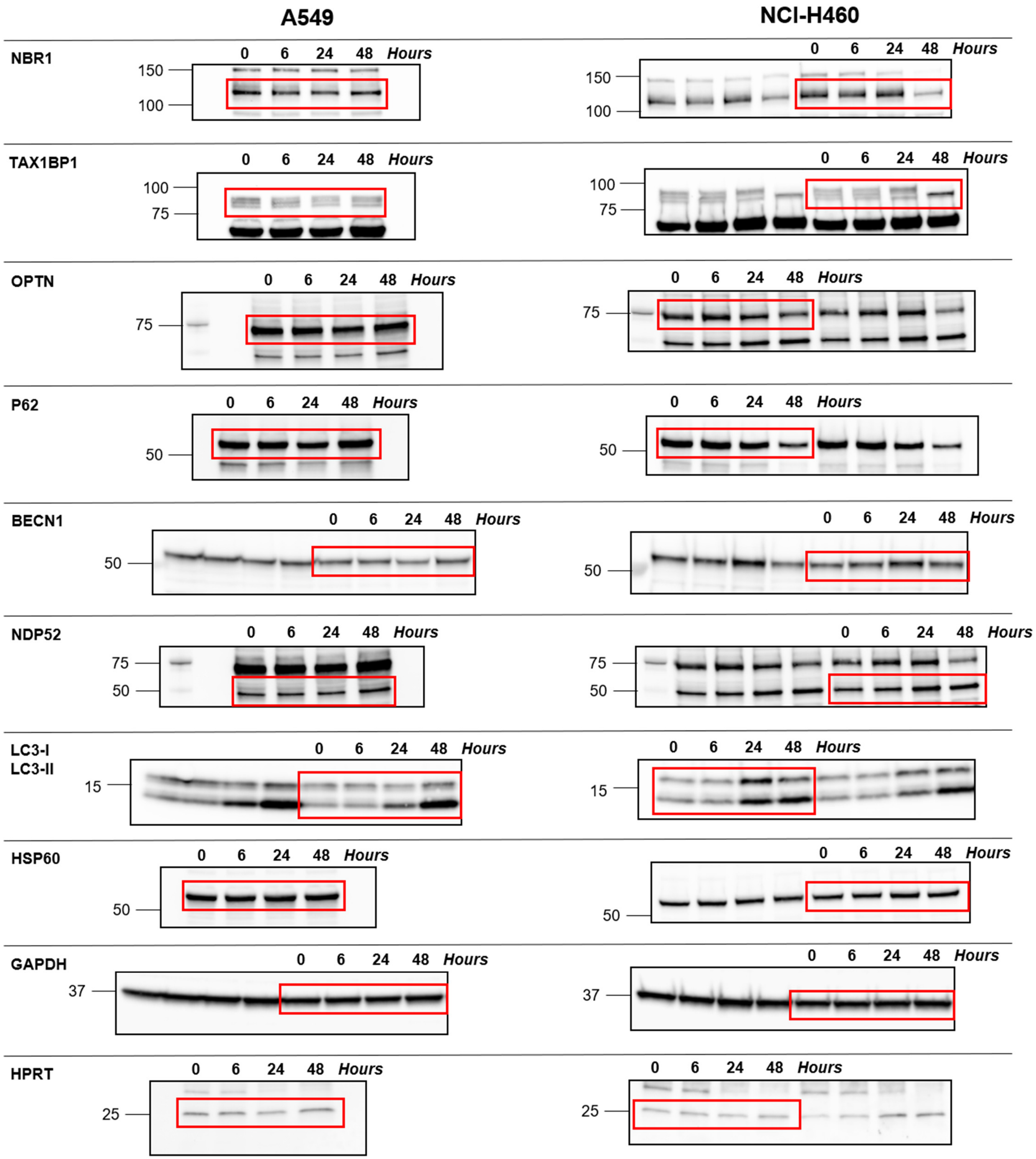

Supplementary Figure 20. Full blots related to Figure 3D.

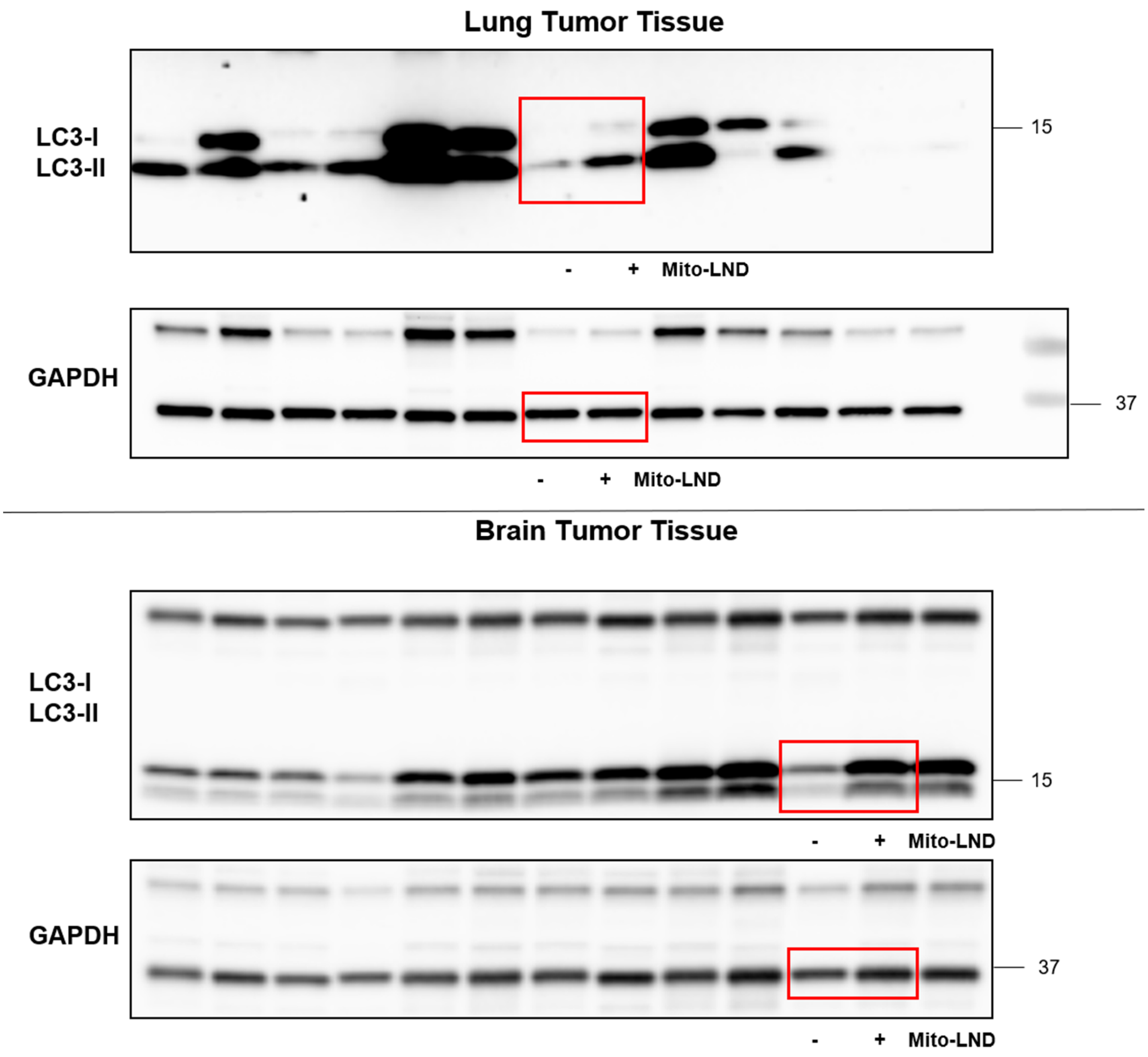

Supplementary Figure 21. Full blots related to Figure 4.

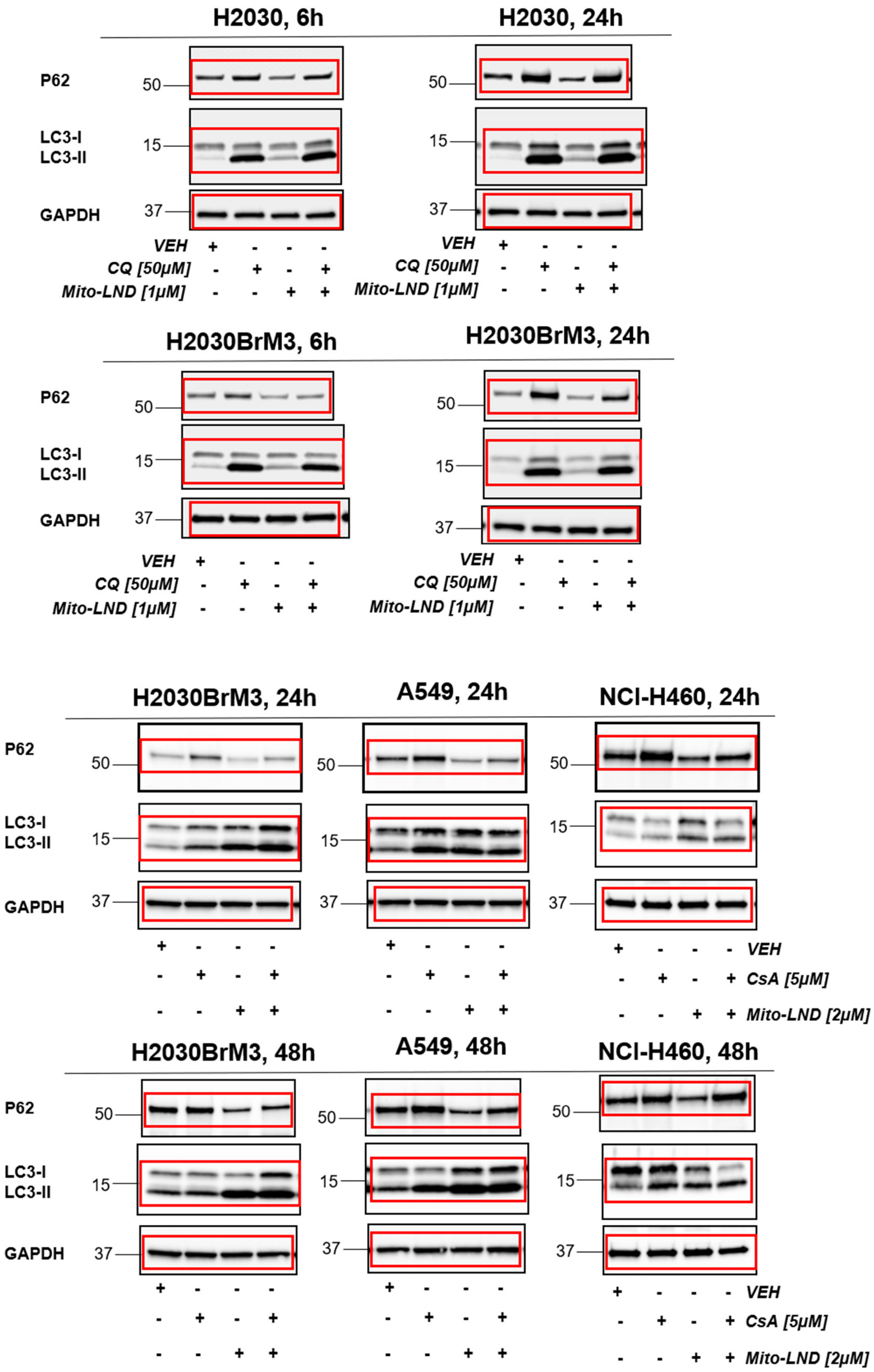

Supplementary Figure 22. Full blots related to Figure 6.

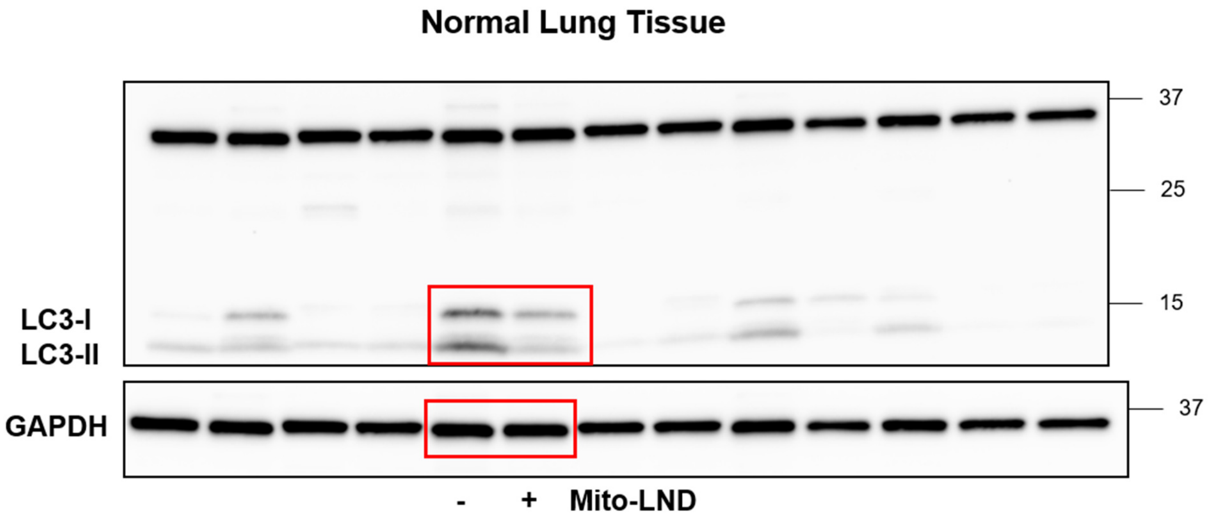

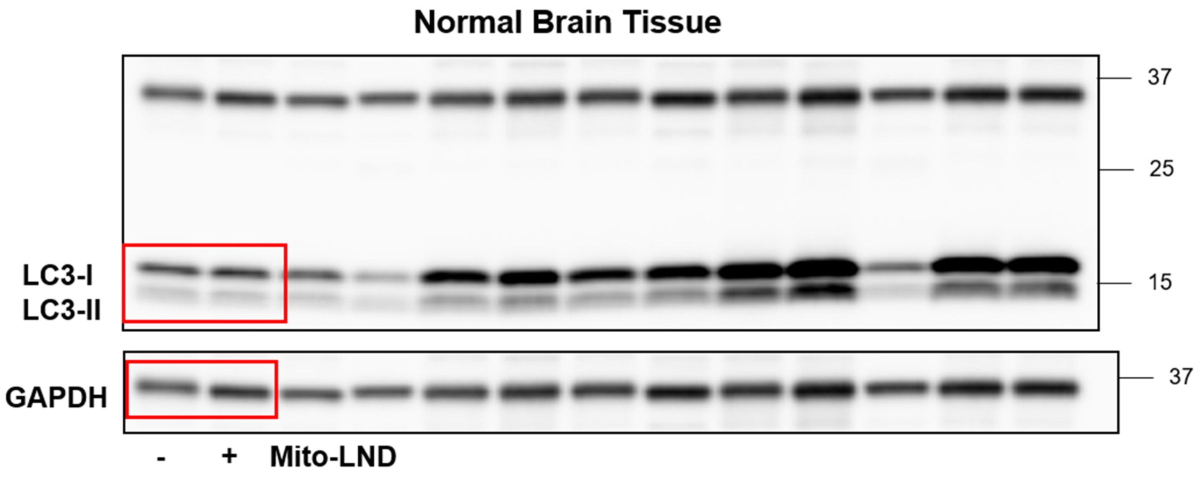

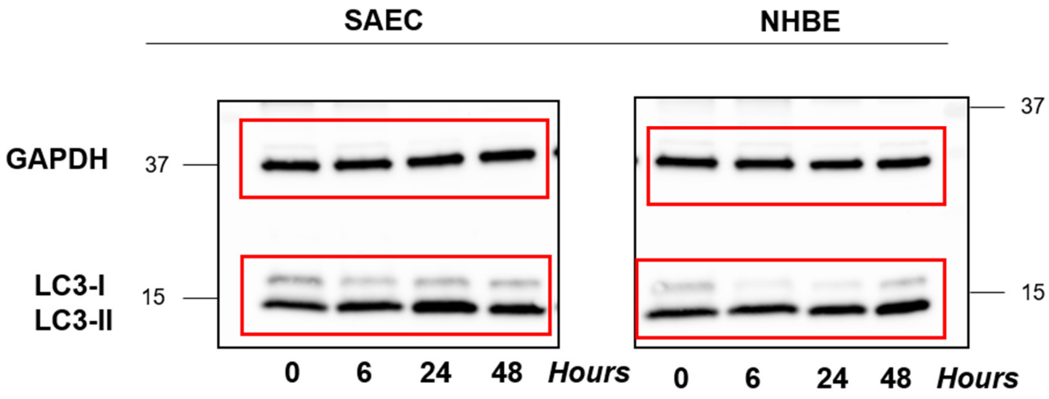

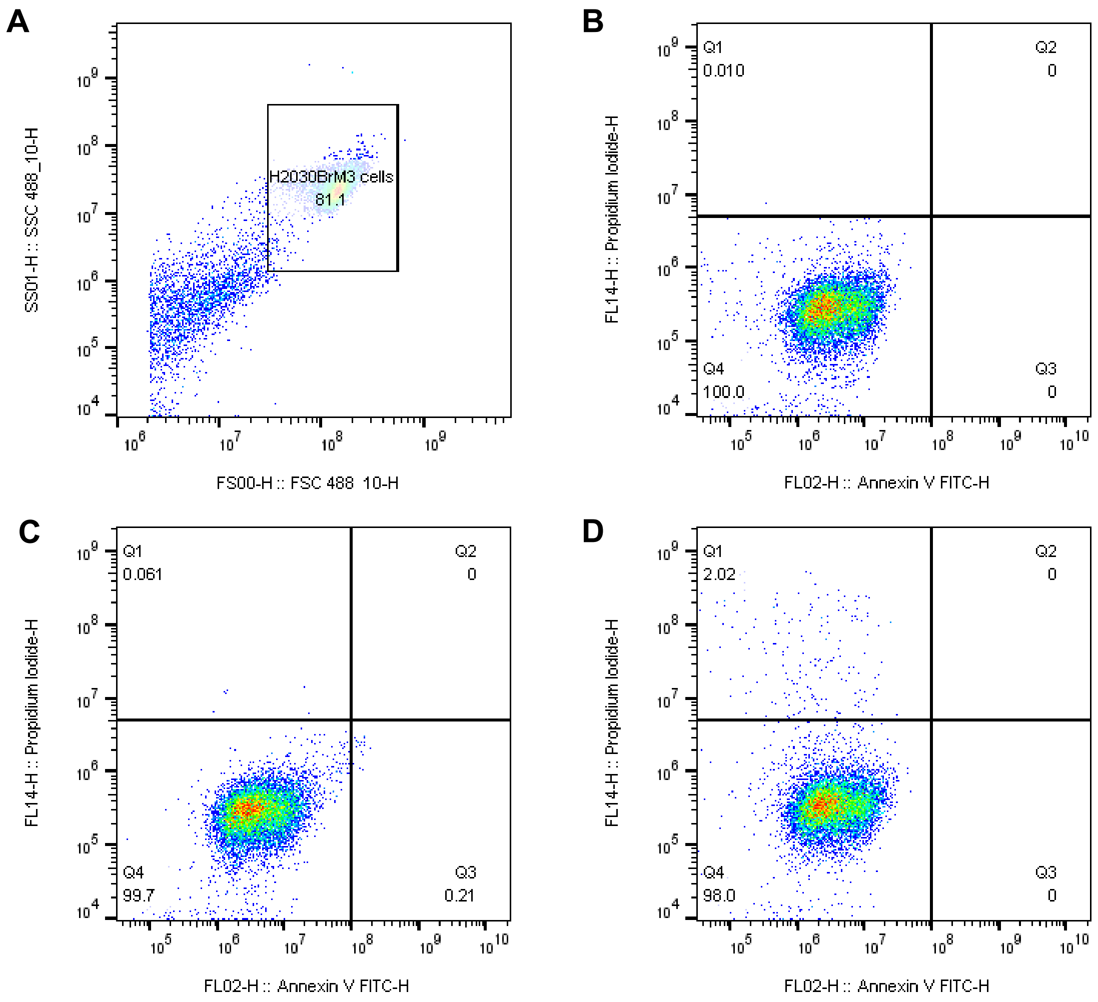

Supplementary Figure 23. Flow cytometry gating strategy. A. Forward scatter (FSC) and side scatter (SSC) profiles were used to identify the H2030BrM3 cell population and exclude debris. **B.** Gating determination based on untreated unstained cells negative for Annexin V FITC and Propidium iodide, Q4. **C.** Positive for Annexin V FITC only, Q3. **D.** Positive for Propidium iodide only, Q1.

Supplementary Table 1. Modified Irwin screen after 8-week Mito-LND treatment

	Control	1x	10x	20x	50x
Physical condition					
Body weight (g)	20.6±0.9	21.0±0.6	22.4±0.6	22.0±0.5	22.4±0.8
Rectal temperature (ºC)	37.6±0.3	37.5±0.3	37.6±0.3	37.5±0.2	37.8±0.3
Presence of whiskers	0.0±0.0	0.0±0.0	0.0±0.0	0.0±0.0	0.0±0.0
Well-groomed	0.0±0.0	0.0±0.0	0.0±0.0	0.0±0.0	0.0±0.0
Piloerection	0.0±0.0	0.0±0.0	0.0±0.0	0.0±0.0	0.0±0.0
Fur missing on face	0.0±0.0	0.0±0.0	0.0±0.0	0.0±0.0	0.0±0.0
Fur missing on body	0.0±0.0	0.0±0.0	0.0±0.0	0.0±0.0	0.0±0.0
Wounds	0.0±0.0	0.0±0.0	0.0±0.0	0.0±0.0	0.0±0.0
Skin color	0.0±0.0	0.0±0.0	0.0±0.0	0.0±0.0	0.0±0.0
Palpebral closure	0.0±0.0	0.0±0.0	0.0±0.0	0.4±0.2	0.0±0.0
Behavior in novel environment					
Transfer behavior	0.0±0.0	0.0±0.0	0.0±0.0	0.0±0.0	0.0±0.0
Body positioning	0.0±0.0	0.0±0.0	0.0±0.0	0.0±0.0	0.0±0.0
Spontaneous activity	0.0±0.0	0.0±0.0	0.0±0.0	0.0±0.0	0.0±0.0
Respiration rate	0.0±0.0	0.0±0.0	0.0±0.0	0.0±0.0	0.0±0.0
Tremor	0.0±0.0	0.0±0.0	0.0±0.0	0.0±0.0	0.0±0.0
Piloerection	0.0±0.0	0.0±0.0	0.0±0.0	0.0±0.0	0.0±0.0
Gait	0.0±0.0	0.0±0.0	0.0±0.0	0.0±0.0	0.0±0.0
Pelvic elevation	2.0±0.0	2.0±0.0	2.0±0.0	2.0±0.0	2.0±0.0
Tail elevation	1.0±0.0	1.0±0.0	1.0±0.0	1.0±0.0	1.0±0.0
Urination	0.4±0.2	0.2±0.2	0.6±0.2	0.4±0.2	0.4±0.2
Defecation	0.4±0.2	0.2±0.2	0.0±0.0	0.4±0.2	0.4±0.2
Reflexes or reaction to stimuli					
Touch escape	0.6±0.2	0.6±0.2	0.8±0.2	0.8±0.2	0.6±0.2
Positional passivity	1.0±0.0	1.0±0.0	1.0±0.0	1.0±0.0	1.0±0.0
Trunk curl	0.0±0.0	0.0±0.0	0.0±0.0	0.0±0.0	0.0±0.0
Reaching reflex	0.0±0.0	0.0±0.0	0.0±0.0	0.0±0.0	0.0±0.0
Pinna reflex	0.6±0.2	1.0±0.0	1.0±0.0	1.0±0.0	1.0±0.0
Preyer reflex at ~90 dB	1.0±0.0	1.0±0.0	1.0±0.0	1.0±0.0	1.0±0.0
Toe pinch response	0.4±0.2	0.4±0.2	0.4±0.2	0.2±0.2	0.6±0.2
Righting reflex	0.0±0.0	0.0±0.0	0.0±0.0	0.0±0.0	0.0±0.0
Air righting reflex	0.0±0.0	0.0±0.0	0.0±0.0	0.0±0.0	0.0±0.0
Inverted screen latency to fall (seconds)	>60.0	>60.0	>60.0	>60.0	>60.0
Provoked biting	1.0±0.0	1.0±0.0	1.0±0.0	1.0±0.0	1.0±0.0
Measures during supine restraint					
Limb tone	0.0±0.0	0.0±0.0	0.0±0.0	0.0±0.0	0.0±0.0
Abdominal tone	0.0±0.0	0.0±0.0	0.0±0.0	0.0±0.0	0.0±0.0
Toe pinch	0.0±0.0	0.0±0.0	0.0±0.0	0.0±0.0	0.0±0.0

Values reflect the mean ± SEM of the deviation score (0=normal, 1=mild deviation, 2=moderate deviation, 3=extreme deviation) or metric noted for each attribute after 8-week treatment with Mito-LND or vehicle. Observations were conducted by two different experimenters and the scores were averaged.
