# Supplementary material for: Targeting lonidamine to mitochondria mitigates lung tumorigenesis and brain metastasis
Source: Nat Commun. 2019 May 17;10:2205. doi: 10.1038/s41467-019-10042-1 (PMC6525201; doi:10.1038/s41467-019-10042-1)
Supplement: Supplementary file 2 — Reporting Summary [file 41467_2019_10042_MOESM2_ESM.pdf]

nature research

Corresponding author(s): Ming You

Last updated by author(s): Apr 5, 2019

Reporting Summary

Nature Research wishes to improve the reproducibility of the work that we publish. This form provides structure for consistency and transparency in reporting. For further information on Nature Research policies, see Authors & Referees and the Editorial Policy Checklist.

Statistics

For all statistical analyses, confirm that the following items are present in the figure legend, table legend, main text, or Methods section.

n/a	Confirmed	
☐	☒	The exact sample size (*n*) for each experimental group/condition, given as a discrete number and unit of measurement
☐	☒	A statement on whether measurements were taken from distinct samples or whether the same sample was measured repeatedly
☐	☒	The statistical test(s) used AND whether they are one- or two-sided<br>*Only common tests should be described solely by name; describe more complex techniques in the Methods section.*
☐	☒	A description of all covariates tested
☐	☒	A description of any assumptions or corrections, such as tests of normality and adjustment for multiple comparisons
☐	☒	A full description of the statistical parameters including central tendency (e.g. means) or other basic estimates (e.g. regression coefficient) AND variation (e.g. standard deviation) or associated estimates of uncertainty (e.g. confidence intervals)
☒	☐	For null hypothesis testing, the test statistic (e.g. *F*, *t*, *r*) with confidence intervals, effect sizes, degrees of freedom and *P* value noted<br>*Give P values as exact values whenever suitable.*
☒	☐	For Bayesian analysis, information on the choice of priors and Markov chain Monte Carlo settings
☒	☐	For hierarchical and complex designs, identification of the appropriate level for tests and full reporting of outcomes
☒	☐	Estimates of effect sizes (e.g. Cohen's *d*, Pearson's *r*), indicating how they were calculated

Our web collection on statistics for biologists contains articles on many of the points above.

Software and code

Policy information about availability of computer code

Data collection	Not applicable, as no custom algorithms or software was used.
Data analysis	Not applicable, as no custom algorithms or software was used.

For manuscripts utilizing custom algorithms or software that are central to the research but not yet described in published literature, software must be made available to editors/reviewers. We strongly encourage code deposition in a community repository (e.g. GitHub). See the Nature Research guidelines for submitting code & software for further information.

Data

Policy information about availability of data

All manuscripts must include a data availability statement. This statement should provide the following information, where applicable:

- Accession codes, unique identifiers, or web links for publicly available datasets
- A list of figures that have associated raw data
- A description of any restrictions on data availability

The data that support the findings of this study are available from the corresponding author upon reasonable request.

Field-specific reporting

Please select the one below that is the best fit for your research. If you are not sure, read the appropriate sections before making your selection.

☒ Life sciences ☐ Behavioural & social sciences ☐ Ecological, evolutionary & environmental sciences

For a reference copy of the document with all sections, see nature.com/documents/nr-reporting-summary-flat.pdf

Life sciences study design

All studies must disclose on these points even when the disclosure is negative.

Sample size	Sample size were calculated based on the detectable differences with at least 80% power, based on a two-sided t-test or chi-square test with a Bonferroni adjusted significance level of 0.05/3=0.017 to protect against multiple testing.
Data exclusions	No exclusion of data.
Replication	All experiments were performed in at least triplicate and all replicates were successful.
Randomization	Animals were randomized to treatment groups based on mean body weight measurements, ensuring no statistical differences between animals at baseline.
Blinding	Technical staff conducting in vivo bioassays and tissue harvests of specimens were aware of group allocations, however, those processing samples and performing laboratory experiments were blinded to group allocations with unblinding following data analysis.

Reporting for specific materials, systems and methods

We require information from authors about some types of materials, experimental systems and methods used in many studies. Here, indicate whether each material, system or method listed is relevant to your study. If you are not sure if a list item applies to your research, read the appropriate section before selecting a response.

Materials & experimental systems

n/a	Involved in the study	
☐	☒	Antibodies
☐	☒	Eukaryotic cell lines
☒	☐	Palaeontology
☐	☒	Animals and other organisms
☒	☐	Human research participants
☒	☐	Clinical data

Methods

n/a	Involved in the study	
☒	☐	ChIP-seq
☐	☒	Flow cytometry
☒	☐	MRI-based neuroimaging

Antibodies

Antibodies used	Immunoblotting was performed using commercially available antibodies from Abcam (Cambridge, MA): NDP52 (#ab68588; 1:500); BD Biosciences (San Jose, CA): P62 (#5114; 1:1000); Cell Signaling Technology (Danvers, MA): AKT (#4691; 1:1000), AMPK (#2532; 1:1000), Bax (#5023; 1:1000), Bcl-2 (#2876; 1:1000), Beclin-1 (#3738; 1:750), Caspase 3 (#9665; 1:500), Caspase 7 (#12827; 1:1000), Caspase 9 (#9508; 1:1000), GAPDH (#2118; 1:40,000), LC3 (#4108; 1:1000), mTOR (#2983; 1:1000), NBR1 (#9891; 1:500), PARP (#9532; 1:500), P70 S6 Kinase (#2708; 1:1000), PTEN (#9552; 1:1000), phospho-AKTSer473 (#4060; 1:1000), phospho-AKTThr308 (#13038; 1:1000), phospho-mTORSer2448 (#5536; 1:500), phospho-P70 S6 KinaseThr389 (#9234; 1:1000), phospho-PTENSer380 (#9551; 1:1000), phospho-ULKSer757 (#6888; 1:1000), RAB7 (#9367; 1:1000), TAX1BP1 (#5105; 1:1000), and ULK (#8054; 1:1000); Novus Biologicals (Littleton, CO): PINK1 (#BC100-494; 1:500); Proteintech Group, Inc. (Rosemont, IL): Optineurin (#10837-1-AP; 1:500); and Santa Cruz Biotechnology (Dallas, TX): Cytochrome c (#sc-13156; 1:500), HSP60 (#sc-13966; 1:5000), Prx1 (Santa Cruz sc-7381); Prx3 (Santa Cruz sc-59661).
Validation	Detailed validation information of these antibodies are available from the websites of Abcam (Cambridge, MA); BD Biosciences (San Jose, CA); Cell Signaling Technology (Danvers, MA); Novus Biologicals (Littleton, CO); Proteintech Group, Inc. (Rosemont, IL); and Santa Cruz Biotechnology (Dallas, TX). All antibodies were properly stored and used by the expiration date.

Eukaryotic cell lines

Policy information about cell lines

Cell line source(s)	H2030 lung adenocarcinoma cells, which have a KRASG12C mutation (25), were purchased from ATCC (catalog #CRL-5914). A549 (ATCC #CCL-185) is a human adenocarcinoma alveolar basal epithelial cell line that has a KRASG12S mutation. NCI-H460 is a human epithelial cell line derived from large cell lung carcinoma tissue (ATCC catalog #HTB-177) and has a KRASQ61H mutation. H2030BrM3 cells (isolated from brain metastases of H2030 cells) were generously provided by Dr. Joan Massagué (Cancer Biology and Genetics Program, Memorial Sloan Kettering Cancer Center, New York, NY). Normal lung cell lines, SAEC and NHBE, were obtained from Lonza (catalog #CC-2540 and #CC-2547, respectively).
Authentication	Cell lines purchased from ATCC and Lonza were stored in liquid nitrogen, and were only used within passage 20.
Mycoplasma contamination	All cell lines tested negative for mycoplasma.

Commonly misidentified lines (See ICLAC register)	N/A

Animals and other organisms

Policy information about studies involving animals; ARRIVE guidelines recommended for reporting animal research

Laboratory animals	A/J mice (both sexes) and NOD/SCID mice (female) of 4 to 8 weeks of age from Jackson Laboratories are used in this study.
Wild animals	N/A
Field-collected samples	N/A
Ethics oversight	All procedures were in accordance with the Medical College of Wisconsin Institutional Animal Care and Use Committee.

Note that full information on the approval of the study protocol must also be provided in the manuscript.

Flow Cytometry

Plots

Confirm that:

☒ The axis labels state the marker and fluorochrome used (e.g. CD4-FITC).

☒ The axis scales are clearly visible. Include numbers along axes only for bottom left plot of group (a 'group' is an analysis of identical markers).

☒ All plots are contour plots with outliers or pseudocolor plots.

☒ A numerical value for number of cells or percentage (with statistics) is provided.

Methodology

Sample preparation	H2030BrM3 (1 × 10^6) were seeded in T-25 flasks (Corning, Thermo Fisher Scientific) and allowed to adhere for 24 h at 37°C in a 5% CO2 atmosphere. Cells were washed treated as described in the Methods section. After 48 h, nonadherent cells were collected by centrifugation and adherent cells were harvested by trypsinization for apoptosis staining. Approximately 5 × 10^5 cells were stained with Annexin V FITC (BD Biosciences) and propidium iodide (BD Biosciences) in 1× Annexin V binding buffer (BD Biosciences) following manufacturer's protocols.
Instrument	Flow cytometric analysis was performed on the Ze5™ cell analyzer (Bio-Rad, Hercules, CA).
Software	Data were collected using the Everest software package (Bio-Rad, Hercules, CA) and analysis was performed using FlowJo software (FlowJo, LLC, Ashland, OR).
Cell population abundance	A minimum of 10^3 H2030BrM3 cells were counted per sample analyzed.
Gating strategy	Forward scatter (FSC) and side scatter (SSC) profiles were used to identify the H2030BrM3 cell population and to exclude debris from the analysis using an unstained sample. Single stain samples (Annexin V FITC only or Propidium iodide only) were used to set the apoptotic gating strategy. Using these gates, we analyzed the percentage of cells in each quadrant that were a) Annexin V-/PI- (live), b) Annexin V+/PI- (early apoptosis), c) Annexin V-/PI+ (necrotic) and d) Annexin V+/PI+ (late apoptosis).

☒ Tick this box to confirm that a figure exemplifying the gating strategy is provided in the Supplementary Information.
